# Rational design of balanced dual-targeting antibiotics with limited resistance

**DOI:** 10.1371/journal.pbio.3000819

**Published:** 2020-10-05

**Authors:** Akos Nyerges, Tihomir Tomašič, Martina Durcik, Tamas Revesz, Petra Szili, Gabor Draskovits, Ferenc Bogar, Žiga Skok, Nace Zidar, Janez Ilaš, Anamarija Zega, Danijel Kikelj, Lejla Daruka, Balint Kintses, Balint Vasarhelyi, Imre Foldesi, Diána Kata, Martin Welin, Raymond Kimbung, Dorota Focht, Lucija Peterlin Mašič, Csaba Pal

**Affiliations:** 1 Synthetic and Systems Biology Unit, Biological Research Center, Szeged, Hungary; 2 University of Ljubljana, Faculty of Pharmacy, Ljubljana, Slovenia; 3 Doctoral School of Theoretical Medicine, University of Szeged, Szeged, Hungary; 4 Doctoral School of Multidisciplinary Medical Sciences, University of Szeged, Szeged, Hungary; 5 MTA-SZTE Biomimetic Systems Research Group, Department of Medical Chemistry, University of Szeged, Hungary; 6 Doctoral School of Biology, Faculty of Science and Informatics, University of Szeged, Szeged, Hungary; 7 HCEMM-BRC Translational Microbiology Lab, Szeged, Hungary; 8 Department of Laboratory Medicine, University of Szeged, Szeged, Hungary; 9 SARomics Biostructures, Medicon Village, Lund, Sweden; Wageningen University, NETHERLANDS

## Abstract

Antibiotics that inhibit multiple bacterial targets offer a promising therapeutic strategy against resistance evolution, but developing such antibiotics is challenging. Here we demonstrate that a rational design of balanced multitargeting antibiotics is feasible by using a medicinal chemistry workflow. The resultant lead compounds, ULD1 and ULD2, belonging to a novel chemical class, almost equipotently inhibit bacterial DNA gyrase and topoisomerase IV complexes and interact with multiple evolutionary conserved amino acids in the ATP-binding pockets of their target proteins. ULD1 and ULD2 are excellently potent against a broad range of gram-positive bacteria. Notably, the efficacy of these compounds was tested against a broad panel of multidrug-resistant *Staphylococcus aureus* clinical strains. Antibiotics with clinical relevance against staphylococcal infections fail to inhibit a significant fraction of these isolates, whereas both ULD1 and ULD2 inhibit all of them (minimum inhibitory concentration [MIC] ≤1 μg/mL). Resistance mutations against these compounds are rare, have limited impact on compound susceptibility, and substantially reduce bacterial growth. Based on their efficacy and lack of toxicity demonstrated in murine infection models, these compounds could translate into new therapies against multidrug-resistant bacterial infections.

## Abstract 2

This study designs novel antimicrobial compounds that inhibit multiple bacterial targets, making the spontaneous evolution of resistance extremely rare. These compounds inhibited the growth of all multidrug resistant Staphylococcus aureus (MRSA) isolates tested, while existing antibiotics remained largely ineffective.

## Introduction

The inappropriate use of antibiotics has selected for the global rise of antibiotic-resistant pathogens, rendering several existing antibiotics ineffective [1]. A potential strategy to overcome this rapid evolution of resistance is antibiotic combination therapy. The rationale for this approach is that the evolution of resistance against 2 antibiotics with different modes of action would require the simultaneous emergence of multiple specific mutations at all targets, which is exceedingly rare [2]. Unfortunately, antibiotic combination–based therapies face several difficulties, including differences in the pharmacodynamics of the component antibiotics [3]. An alternative possibility is the the design of antimicrobial compounds that equipotently inhibit multiple bacterial targets [4,5]. There are multiple potential ways to design such compounds. Hybrid drugs consists of 2 covalently linked antibiotic pharmacophores with different molecular targets [6]. Other antibiotics target 2 or more nonoverlapping regions of a single bacterial protein [2] and furthermore, equipotently inhibit 2 or more bacterial proteins. Although it is a major focus of the pharmaceutical industry [4,5,7], designing multitargeting antibiotics remains challenging. Indeed, only a handful of such antibiotic candidates display a balanced inhibition at multiple microbial targets [8–11].

In the current study, we have aimed to rationally develop a new chemical class of antibacterial compounds against multiple, well-established molecular targets that simultaneously fulfill the following criteria: First, the new compounds should display balanced multitargeting activity against multiple, essential bacterial targets. Second, they should establish strong intermolecular interactions at multiple, functionally essential amino acid positions within the binding sites of their target proteins [7]. Such interactions are hypothesized to render spontaneous resistance acquisition improbable because mutations at these sites would compromise the functionalities of the target proteins.

Bacterial DNA gyrase and topoisomerase IV protein complexes offer an exceptional opportunity to achieve this goal, because of the homology of their corresponding subunits and the substantial overlap in their 3-dimensional protein structures [4,12,13]. Both DNA gyrase and topoisomerase IV are heterotetramers, with 2 subunits, GyrA-GyrB and ParC-ParE, respectively. They are involved in breaking and rejoining double-stranded DNA, and thus, they determine changes in DNA topology, but the 2 complexes have complementary roles. DNA gyrase is essential for the negative supercoiling of DNA at the expense of ATP hydrolysis, whereas topoisomerase IV is responsible for unlinking or decatenating DNA following DNA replication [13]. These 2 complexes are clinically validated antibacterial targets: A substantial fraction of antibiotics currently used in clinical settings are inhibitors of bacterial DNA gyrase or topoisomerase IV enzymes [12–15]. Unfortunately, clinically significant resistance against fluoroquinolones and other frequently employed DNA gyrase inhibitors has already arisen in pathogenic bacteria, partly due to the step-by-step accumulation of resistance-conferring mutations at the genes encoding their target proteins [16,17]. This is not unexpected, for 2 reasons. First, fluoroquinolones do not target the gyrase and topoisomerase complexes equipotently [10,11,16,17]. Second, low level of resistance against these antibiotics can readily emerge by mutations in efflux pumps and transcription-translation [18]. Even worse, fluoroquinolone resistance mutations also promote the acquisition of resistance in other antibiotic candidates currently under clinical development [8,19–20].

In the current work, we have rationally designed a novel series of antibacterial compounds, endeavoring to achieve a balanced and simultaneous inhibitory effect on subunit B of DNA gyrase and subunit E of topoisomerase IV. There have been prior studies in this direction, but their clinical relevance is questionable, for at least 1 of the following 4 reasons. They generally failed to achieve equipotent inhibition of both target proteins [19–20], antibacterial acitvity was relatively low, or in vivo infection/toxicity assays were missing or inconclusive [21–24]. Moreover, they generally do not provide detailed resistance analysis, or the resulting lead compound has remained prone to resistance [8].

To target an unmet medical need, we focused on developing antibiotic leads against gram-positive pathogens with a primary focus on methicillin-resistant (MRSA) and vancomycin-intermediate (VISA) *Staphylococcus aureus* isolates [1,25–28]. Multidrug-resistant *S*. *aureus* infections pose an immense economic burden, corresponding to a total of $4.84 billion in annual hospitalization costs [29,30]. Concerns regarding the appropriateness of linezolid, delafloxacin, and other recently marketed “last resort” antistaphylococcal antibiotics have also emerged, not least because resistance has already been detected against these antibiotics in clinical isolates [8, 31–34]. For these reasons, developing a novel class of molecules with a distinct mode-of-action is of utmost importance [35,36].

In this work, we designed balanced multitargeting antibiotics with limited resistance potential. We demonstrate the biochemical and antibacterial characteristics, as well as the in vivo efficacy of 2 representative compounds, termed ULD1 and ULD2.

### Rational design of dual-targeting antibiotics with multiple interacting residues

We have recently discovered a novel class of DNA gyrase inhibitors with a pyrrolamidobenzothiazole scaffold, inspired by the marine natural product oroidin [37–38]. Most compounds from the published series primarily act on the bacterial DNA gyrase complex only and possess weak antibacterial activity. To transform these molecules into broadly effective antibiotics, we have executed modifications at several sites on the pyrrolamidobenzothiazole-6-carboxylic acid scaffold. Thanks to the availability of the co-crystal structure with subunit B of DNA gyrase, as well as to the small size of the molecule and its straightforward chemical synthesis, we have based our efforts on ULD0, a recently reported weak inhibitor of *S*. *aureus* DNA gyrase [37]. The molecular modifications were aimed at designing novel inhibitors that display equipotent dual-targeting activity towards subunit B of DNA gyrase (GyrB) and subunit E of topoisomerase IV (ParE), by a simultaneous establishment of strong interactions with multiple, functionally essential amino acids of both target proteins.

**Fig 1:**
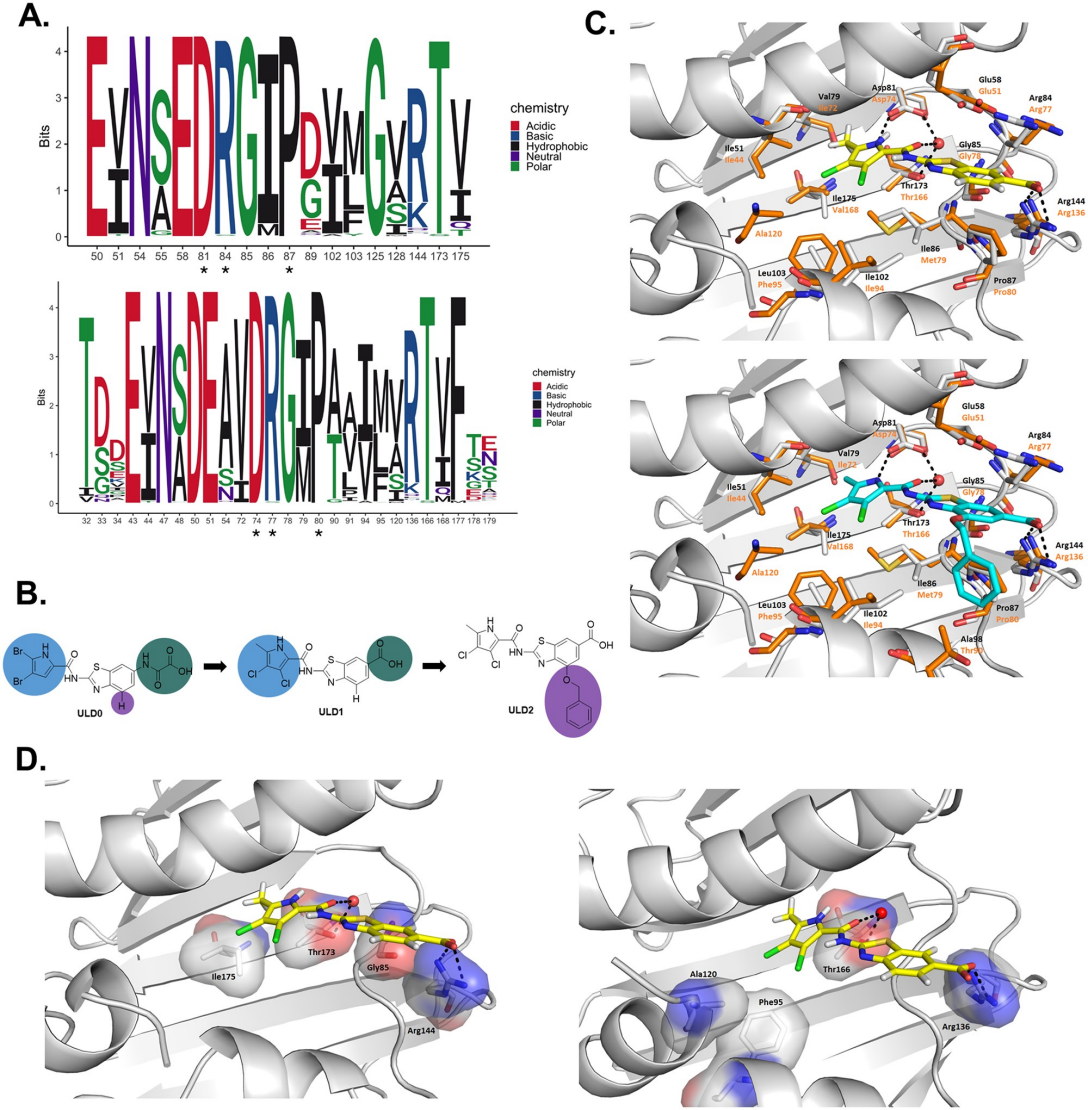
Rational design of antibacterial compounds, ULD1 and ULD2. We rationally designed antibacterial compounds, ULD1 and ULD2, that establish intermolecular interactions with Asp81/Asp74, Arg84/Arg77, and Pro87/Pro80 of *S*. *aureus* GyrB/ParE, respectively. **A.** Amino acid positions that are indicated by an asterisk are phylogenetically more conserved than others in the ATP-binding sites of DNA gyrase subunit B (GyrB, upper panel) and topoisomerase IV subunit E (ParE, lower panel) (Welch *t*-test, *P* < 0.0017 and *P* < 0.00001, respectively). The sequence logo depicts the diversity of the aligned sequences: The relative sizes of the letters indicate their frequency in the sequences. The total height of the letters depicts the information content of the position, in bits. **B.** Rational design of ULD1 and ULD2 as DNA GyrB and ParE dual-inhibitors. The green, blue, and purple circles indicate the terminal carboxylic acid moiety, the pyrrole moiety, and the benzyloxy substituent at position 4 of the benzothiazole ring, respectively, which were modified to obtain ULD1 and ULD2. **C.** The interaction pattern of ULD1 (in yellow, top) and ULD2 (in cyan, bottom) within the ATP-binding site of *S*. *aureus* GyrB and ParE. GyrB and ParE amino acids are indicated in black and orange (with sticks), respectively. PDB codes: GyrB (3TTZ), ParE (4URN). **D.** The location of spontaneous ULD1 resistance-conferring mutations within the ULD1 binding pocket of GyrB (left panel) and ParE (right panel), respectively. Resistance-conferring mutations were identified based on targeted single-molecule real-time sequencing of the drug targets following a standard frequency-of-resistance assay, and the identified mutations were subsequently plotted on the tertiary structure of the target proteins. Atom-type coloring: blue for N, red for O, gray for C. GyrB, subunit B of DNA gyrase; ParE, subunit E of topoisomerase IV.

To this aim, we have focused on 3 key amino acid residues within the ATP-binding sites of GyrB and the corresponding homologous amino acid residues of ParE (Asp81/Asp74, Arg84/Arg77 and Pro87/Pro80). Bioinformatic analyses have revealed a very limited variation at these amino acid positions across over 1,000 phylogenetically diverse bacterial genomes, including representative species belonging to Actinobacteria, Firmicutes, Bacteroidetes, Proteobacteria, Chlamydiae bacterial phyla. These amino acid residues were 99% to 100% conserved in the studied genomes (Fig 1A). Moreover, using site-directed mutagenesis, a previous study showed that these 3 amino acids are essential for the enzymatic function of GyrB in *Escherichia coli* [39]. Accordingly, we have developed novel compounds that form (1) a hydrogen bond with the Asp81/Asp74 side-chain carboxylate group, (2) a cation-π interaction with the Arg84/Arg77 side-chain guanidinium group, and (3) additional hydrophobic interactions with Pro87/Pro80 of *S*. *aureus* GyrB/ParE, respectively (Fig 1B and 1C, S1 and S2 Tables). Moreover, we have enhanced the compounds’ binding affinity by simultaneously establishing a salt bridge with Arg144/Arg136 and additional hydrophobic interactions within the lipophilic floor of the binding sites on both target proteins (Fig 1B and 1C).

Chemical modifications of ULD0 have yielded 2 antibiotic leads, ULD1 and ULD2. Analysis of the solved crystal structure of ULD1 and ULD2 in complex with *S*. *aureus* GyrB (Fig 1D, PDB entry: 6TCK), as well as molecular dynamics (MD) simulations have shown that the antibiotic leads interact with multiple amino acid residues within the ATP-binding sites of GyrB and ParE, including the ones mentioned here (Fig 1D, S1A and S1B Fig). In the next sections, we demonstrate that these 2 lead compounds are potent dual-targeting enzyme inhibitors and display a broad-spectrum activity against multiple gram-positive human pathogens.

### Improved and balanced enzyme inhibition in vitro

Inhibitory activities of ULD1 and ULD2 on *S*. *aureus* DNA gyrase and topoisomerase IV have been tested in an in vitro gel-based supercoiling assay [40] (Table 1). Novobiocin and ciprofloxacin, 2 inhibitors of bacterial type II topoisomerases, were used as controls. Ciprofloxacin displayed a weak inhibitory effect on these 2 enzymes, whereas novobiocin efficiently blocked DNA gyrase only. In sharp contrast, ULD1 and ULD2 potently inhibited both DNA gyrase and topoisomerase IV. ULD2 inhibited both enzymes to a greater extent than ULD1, with IC_50_ values being 4-fold (DNA gyrase) and 5-fold (topoisomerase IV) lower than those for ULD1. Based on these in vitro enzyme data, we conclude that ULD1 and ULD2 are potent dual-inhibitors of DNA gyrase and topoisomerase IV of *S*. *aureus*, active in the low nanomolar range (Table 1).

**Table 1:** In vitro inhibition of DNA gyrase and topoisomerase IV by ULD1 and ULD2.

	IC_50_				
	ULD0*	ULD1	ULD2	Novobiocin	Ciprofloxacin
DNA gyrase	>100 μM	3.3 ± 0.4 nM	0.78 ± 0.1 nM	1.7 ± 0.1 nM	14,000 ± 2,000 nM
Topoisomerase IV	10 ± 2 μM	9.3 ± 2.2 nM	2.0 ± 0.3 nM	2,000 ± 200 nM	1,500 ± 300 nM

Results are based on standard *S*. *aureus* DNA gyrase and topoisomerase IV supercoiling gel-based assays (Materials and methods). Novobiocin and ciprofloxacin were applied as comparator antibiotics. Measurements were performed in quadruplicates (mean and standard deviation of the mean are shown).

*ULD0 was tested using the high-throughput DNA gyrase supercoiling and topoisomerase IV relaxation assays [37].

IC_50_, half maximal inhibitory concentration.

### Bioactivity of ULD1 and ULD2 against pathogenic bacteria

Next, we have determined the minimum inhibitory concentrations (MICs) of ULD1 and ULD2 against a panel of gram-negative and gram-positive clinical pathogens (Table 2 and S3 Table). ULD1 and ULD2 were found to display potent antibacterial activity against ESKAPE pathogens (*S*. *aureus*, *Enterococcus* sp., *Pseudomonas aeruginosa*, *Klebsiella pneumoniae*, *Acinetobacter baumannii)*, *Streptococcus* sp., and *Clostridium difficile*. The MIC values against all studied multidrug-resistant *Staphylococcus*, *Enterococcus*, and *Streptococcus* isolates were below 2 μg/mL. Notably, ULD1 and ULD2 exerted activity against all MRSA, VRSA, and vancomycin-resistant *Enterococcus* (VRE) isolates, which frequently cause difficult-to-treat skin and soft-tissue infections (SSTIs) [41]. We hypothesize that further chemical modifications of ULD1/ULD2 could increase the potency of this compound class to inhibit gram-negative pathogens as well.

**Table 2:** Antimicrobial activities of ULD1 and ULD2 against selected pathogenic bacteria.

Species and strain	Acquired resistance	ULD1	ULD2
		MIC (μg/mL)	
*S*. *aureus* ATCC 700699 (Mu50, NRS1)	MRSA (SCC*mec* type II), VISA, Clindamycin-R, Daptomycin-NS, Erythromycin-R, Gentamycin-R, Imipenem-R, Levofloxacin-R, Oxacillin-R	0.0625	≤0.03125
*S*. *aureus* ECL 2963646	MRSA, VRSA, Clindamycin-R, Erythromycin-R, Gentamycin-R, Levofloxacin-R	0.125	≤0.03125
*Staphylococcus epidermidis* ATCC 51625	MRSE, Oxacillin-R	0.0625	≤0.03125
*Enterococcus faecalis* ATCC 51575	VRE (VanB+), Clindamycin-R, Erythromycin-R, Gentamycin-R, Linezolid-IR, Mupirocin-R, Streptomycin-R	0.0625	≤0.03125
*Enterococcus faecium BAA-2320*	VRE (VanA+), Ampicillin-R, Ciprofloxacin-R, Clindamycin-R, Erythromycin-R, Levofloxacin-R, Imipenem-R, Teicoplanin-R	0.25	≤0.03125
*Neisseria gonorrhoeae* CCUG 57598	Cefoxitin-R, Ciprofloxacin-R, Linezolid-R, Tetracycline-R, Penicillin-R	≤0.03125	≤0.03125
*Haemophilus influenzae* ATCC 49247	Ampicillin-R, Vancomycin-R, Tetracycline-R	≤0.03125	0.125
*Clostridium difficile* BAA-1875	toxigenic, ribotype 078, Ertapenem-IR, Imipenem-R	≤0.03125	≤0.03125
*Listeria monocytogenes* ATCC 19111		0.125	0.125
*Acinetobacter baumannii* ATCC 19606		2	0.5
*Klebsiella pneumoniae* ATCC 10031		1	4
*Pseudomonas aeruginosa* ATCC 27853		8	2

MIC measurements were performed in 3 replicates according to CLSI guidelines.

-R, resistant; -IR, intermediate resistant; -NS, nonsensitive; CLSI, Clinical and Laboratory Standards Institute; MIC, minimum inhibitory concentration; MRSA, methicillin-resistant *S*. *aureus*; MRSE, methicillin-resistant *S*. *epidermidis*; VISA, vancomycin-intermediate *S*. *aureus*; VRE, vancomycin-resistant *Enterococcus*; VRSA, vancomycin-resistant *S*. *aureus*.

**Fig 2:**
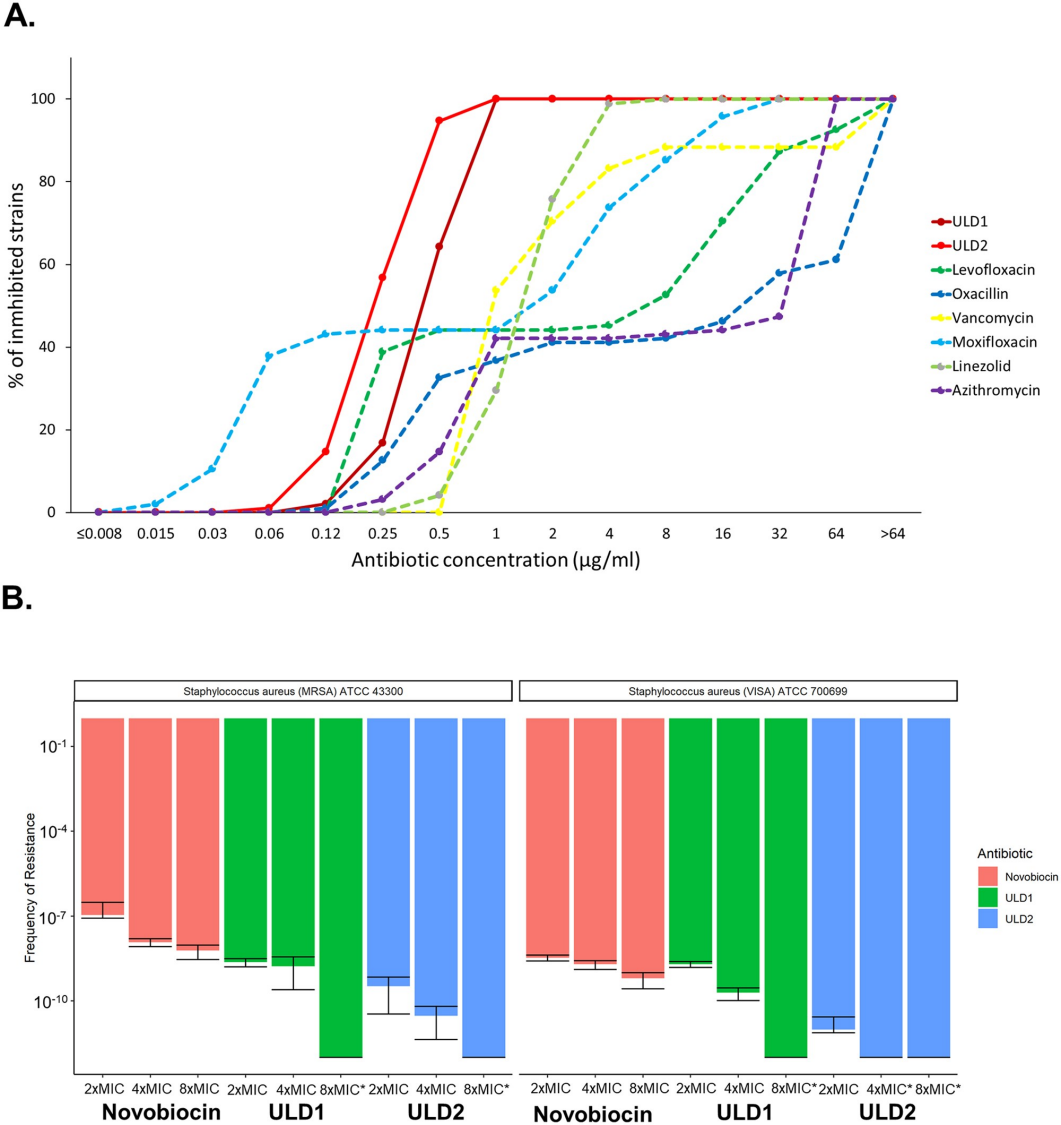
Antibacterial activities and spontaneous frequency-of-resistance of ULD1 and ULD2. **A.** Antibacterial activities of ULD1 and ULD2 against a panel of 95 *S*. *aureus* clinical isolates. Strains included 55 MRSA and 28 vancomycin-intermediate or -resistant isolates with diverse geographic origins (see S1 Data Table 1 for the strains’ description). All strains (100%) were inhibited at 1 μg/mL concentration of ULD1 and ULD2. MICs were determined via broth microdilution according to CLSI guidelines. The underlying data for this figure can be found in S2 Data. **B.** Spontaneous frequency-of-resistance of ULD1, ULD2, and novobiocin in *S*. *aureus* MRSA ATCC 43300 (left panel) *S*. *aureus* VISA ATCC 700699 (right panel). Data are based on 10 independent biological replicates. Error bars indicate the 95% confidence interval. Asterisk (*) marks cases where the frequency-of-resistance was below 1×10^−12^. The underlying data for this figure can be found in S2 Data. CLSI, Clinical and Laboratory Standards Institute; MIC, minimum inhibitory concentration; MRSA, methicillin-resistant *S*. *aureus*; VISA, vancomycin-intermediate *S*. *aureus*.

We have focused on determining the antibacterial activity of ULD1 and ULD2 against a geographically and genetically diverse set of *S*. *aureus* clinical isolates, including 56 MRSA and 28 vancomycin-intermediate and vancomycin-resistant strains, inclusive of recent clinical isolates. A large fraction of these isolates were simultaneously resistant to multiple other available antibiotics too (S1 Data). In sharp contrast to other approved antibiotics against staphylococcal infections, both ULD1 and ULD2 were found to exert a potent activity against all tested isolates (MIC ≤ 1 μg/mL, Fig 2A). The compounds were also tested in a time-dependent cell killing assay against *S*. *aureus* ATCC 700699 (VISA). The cell killing (bactericidal) activity of ULD1 and ULD2 was demonstrated to exceed that of fusidic acid (S2 Fig). Importantly, regrowth was observable in *S*. *aureus* populations within 48 hours under fusidic acid stress, possibly explained by the fact that *S*. *aureus* rapidly develops resistance to fusidic acid due to its monotargeting mechanism-of-action [4, 42].

### Increased bioactivity at acidic pH of the skin

*S*. *aureus* is a common cause of severe and difficult-to-treat skin infections [41,43]. Antibiotics targeting pathogens specific to skin infections must have an efficient bactericidal effect when applied topically. Compared with systemic antibacterial therapy when the pharmaceutical agent is supposed to exert its activity at physiological blood pH (i.e., 7.35–7.45), topical antimicrobial therapy should typically be active at lower (acidic) pH values characteristic of the skin surface (i.e., 4.0–5.5) [44,45]. Thus, for topical antistaphylococcal agents, maintaining bioactivity under acidic pH conditions is an important feature [46,47]. To assess the effects of pH on our ULD agents’ bioactivity, we have repeated our previous MIC measurements at a lower pH. A shift from pH 7.3 to 5.5 has resulted in a decrease of MIC values as high as 160-fold (ULD1) and 40-fold (ULD2) in *S*. *aureus* ATCC 700699 (S4 Table). Under the same conditions, the potency of vancomycin has been found to be unaffected. We speculate that this increased bioactivity of ULD1 and ULD2 under acidic pH conditions may reflect an increased intracellular accumulation of these compounds within bacterial cells at acidic pH, similarly to delafloxacin [48]. As increased bioactivity at lower-than-neutral pH is beneficial for the treatment of staphylococcal infections [47,48], our newly developed ULD agents could offer especially powerful alternatives for the eradication of *S*. *aureus* in acidic environments, including the human skin surface and macrophages.

### Limited resistance against ULD1 and ULD2

Antibiotics with a single molecular target are usually prone to resistance acquisition induced by spontaneous mutations. Multitargeting antibiotics are considered to be less vulnerable to resistance, as the simultaneous acquisition of multiple, specific mutations is exceedingly rare [4, 49, 50]. To explore the potential resistance mechanisms, we have determined the spontaneous frequency-of-resistance against ULD1, ULD2, and novobiocin in *S*. *aureus*. Novobiocin has served as a reference compound. Novobiocin’s main target is the DNA gyrase subunit B, but second-step resistance mutations occasionally occur in topoisomerase IV [51]. This antibiotic is effective against certain gram-positive infections, including those caused by *S*. *aureus* [12, 52].

Using a standard protocol for spontaneous frequency-of-resistance analysis [49, 53], we have exposed 10^10^ to 10^12^ bacterial cells derived from stationary-phase cultures of *S*. *aureus* ATCC 700699 (VISA) and *S*. *aureus* ATCC 43300 (MRSA) to increasing concentrations of ULD1, ULD2, and novobiocin, respectively. We have assessed the frequency-of-resistance and the mutant prevention concentration (MPC) for all 3 compounds. MPC is the drug concentration threshold above which the selective proliferation of resistant mutants does not occur (i.e., the concentration required to avoid the emergence of all first-step resistant mutants) [49]. In agreement with prior laboratory studies and clinical observations, the frequency-of-resistance against novobiocin was relatively high (Fig 2B), and an up-to 120-fold increment in the MIC of novobiocin in the isolated *S*. *aureus* mutants have been detected (S3 Fig) [51,54]. By sharp contrast, no resistant variants of *S*. *aureus* have been detected when the bacterial cells were exposed to ULD1 at concentrations 8-fold the wild-type MIC (Fig 2B). Moreover, resistant mutants isolated upon exposure to ULD1 at lower compound concentrations provided only minor changes in ULD1 susceptibility (S3 Fig).

To investigate the molecular basis of mild ULD1 resistance, we have collected 400 independently isolated, ULD1 resistant clones from the frequency-of-resistance assay plates and have sequenced their *gyrB* and *parE* genomic regions using Pacific Biosciences single-molecule real-time (SMRT) amplicon sequencing. Sequence analyses have revealed that all ULD1-resistant isolates had missense mutations that mapped to *gyrB*. Four different positions in the ULD1-binding pocket of GyrB (R144, G85, I175, T173) have mutated repeatedly (S5 Table). All the mutated amino acid residues in *S*. *aureus* are located in the binding pocket of GyrB and form secondary interactions with ULD1 (S2 Table and S1B Fig).

As ULD2 has a high affinity towards both of its target proteins and exerts an excellent dual-target enzyme inhibition (Table 1), we hypothesized that the frequency-of-resistance against ULD2 could be exceptionally low. Notably, no ULD2 resistant mutants have emerged when 4×10^12^ *S*. *aureus* ATCC 700699 (VISA) cells were exposed to ULD2 at a concentration of only 4 times the wild-type MIC (Fig 2B). We estimate that the MPC is as low as 0.16 μg/mL for ULD1 and 0.08 μg/mL for ULD2 in *S*. *aureus* ATCC 700699 (VISA).

### Resistance induced by mutations at both target proteins

The frequency-of-resistance assays have indicated that spontaneous resistance evolution to ULD1 and ULD2 is rare and is responsible for only a modest decrease in compound susceptibility of *S*. *aureus*. However, a prior study suggests that combination of multiple, specific mutations at all drug targets, in the long run, can eventually render even multitargeting antibiotics ineffective [8]. To test this possibility, we have repeated the frequency-of-resistance assays with 2 ULD1-resistant *S*. *aureus* VISA laboratory isolates, both of which carried a single mutation at GyrB, the primary target of ULD1/ULD2. These mutations—GyrB R144I and I175T—were relatively frequent in the isolated single-step resistant mutants, and they conferred a decreased susceptibility to ULD1 (S6 Table and S3 Fig). Populations of these single-mutant strains have been exposed to increasing concentrations of ULD1 and ULD2, separately. Spontaneous resistant mutants have appeared at a frequency of 10^−8^–10^−11^ (S4A and S4B Fig). All isolated second-step resistant mutants have displayed a relatively low resistance level, i.e., they could be inhibited by 1 μg/mL of ULD2 (S6 Table). Sequence analyses have revealed that all detected second-step mutations are localized at ParE, the other target of ULD1/ULD2, at positions that are homologous to the binding sites in GyrB (S6 Table). In sum, the observed mutations, together with results of in vitro enzyme inhibition assays, provide strong evidence in support of a dual-targeting mechanism-of-action for ULD1 and ULD2.

### Evolution of resistance under long-term antibiotic exposure

**Fig 3:**
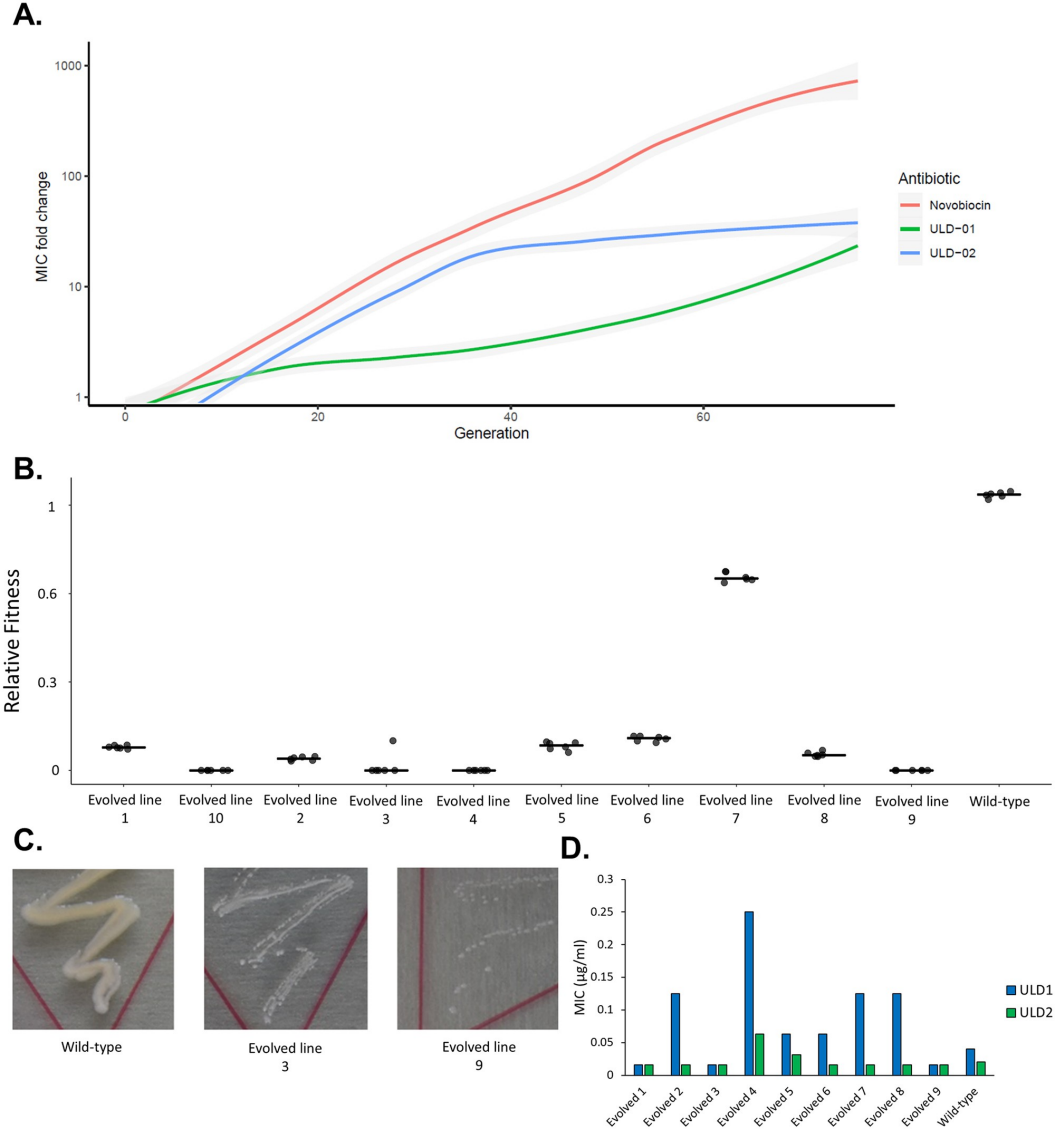
Adaptive laboratory evolution of *S*. *aureus* (VISA) ATCC 700699 to ULD1, ULD2, and novobiocin stresses. **A.** The figure displays increment in MIC level relative to wild type as a function of cell generation number. Data show the mean MIC fold-change based on 10, independently evolving populations. Gray area represents a 95% confidence interval. The underlying data for this figure can be found in S2 Data. **B.** and **C.** Relative fitness (**B**) and growth phenotype (**C**) of ULD1-evolved and wild-type *S*. *aureus* VISA ATCC 700699. Fitness was approximated from the growth curves of isogenic microbial populations (see Materials and methods) and depicted as relative fitness compared with that of the wild type. Measurements were performed in 6 replicates. The underlying data for this figure can be found in S2 Data. Growth phenotypes were observed in BHI agar plates and documented after 24 hours of incubation at 37 °C. **D.** Susceptibility of novobiocin-resistant *S*. *aureus* VISA ATCC 700699 mutants to ULD1 and ULD2. MICs were determined in MHBII medium at 37 °C by broth microdilution assay according to CLSI guidelines. One of the independently evolved novobiocin-adapted strains displayed exceedingly slow growth, and therefore, it was omitted from the analysis. The underlying data for this figure can be found in S2 Data. BHI, Brain-Heart-Infusion broth; CLSI, Clinical and Laboratory Standards Institute; MIC, minimum inhibitory concentration; MHBII, Mueller Hinton II Broth; MRSA, methicillin-resistant *S*. *aureus*; VISA, vancomycin-intermediate *S*. *aureus*.

We have investigated whether long-term exposure to ULD1 and ULD2 could select for a high level of resistance. To this aim, we have initiated adaptive laboratory evolution experiments under ULD1, ULD2, and novobiocin stresses against VISA. We have employed a previously established protocol that aims to maximize the level of drug resistance in the evolving bacterial populations [8,55]. To accurately assess potential resistance mechanisms, 10 parallel evolving populations have been exposed to gradually increasing concentrations of each compound. Following laboratory evolution, a single clone from each population has been isolated and subjected to drug susceptibility tests. In agreement with prior clinical observations and laboratory studies [12,54], a high level of novobiocin-resistance has emerged rapidly (Fig 3A). In novobiocin-adapted strains, an up-to-320-fold increase in novobiocin MIC (i.e., 16 μg/mL) has been detected, compared with the wild-type strain. In contrast, only a relatively modest, 25-fold increase in the MICs of ULD1 and ULD2 have been detected in lineages adapted to ULD1 or ULD2, respectively (1 μg/mL for ULD1 and 0.5 μg/mL for ULD2). In order to elucidate the molecular mechanisms underlying ULD2 resistance, 5 ULD2-adapted strains have been subjected to whole-genome sequencing. We have focused on de novo mutations that have accumulated in several lineages independently during the course of laboratory evolution (S7 Table). Such mutations have been found in the target proteins (GyrB, ParE), as well as in a regulator of purine biosynthesis (*purR*), and another enzyme involved in the uridine diphosphate (UDP) biosynthesis pathway (PyrH). The exact roles of these mutations in shaping ULD1/ULD2 susceptibilities remain to be discovered.

Antibiotic resistance mutations frequently impact bacterial viability, and the associated fitness costs determine the spread and long-term maintenance of resistant populations in clinical settings [55–57]. To explore the potential costs of resistance, we have investigated the growth phenotype of laboratory evolved, ULD1/ULD2 resistance-conferring *S*. *aureus* VISA isolates. Fitness was estimated by measuring the optical density at 600 nm (OD_600_) of the population during 48 hours of growth in an antibiotic-free medium. ULD1/ULD2 resistant clones displayed a statistically significant deterioration of growth pattern compared with the wild-type strain (Fig 3B and S5A Fig) and formed tiny, slow-growing colonies on agar plates (Fig 3C and S5B Fig). These data indicate that long-term exposure to ULD1 and ULD2 yields mutants with limited resistance and high associated fitness costs in an antibiotic-free environment.

Extensive antibiotic usage can select for mutations that provide cross-resistance to antimicrobial compounds that are still under development [8, 58]. As novobiocin was widely employed and is prone to resistance evolution [12, 42], it is rational to hypothesize that novobiocin-resistant clinical isolates might interfere with the antibacterial effects of ULD1 and ULD2. To investigate potential cross-resistance, the MIC of ULD1 and ULD2 have been tested against 9 independently evolved novobiocin-resistant isolates. These strains have been found to display no cross-resistance to ULD2, and only a modest, up to 6-fold decrease in ULD1 susceptibility has been detected compared with the corresponding wild-type strain (Fig 3D).

### Toxicology studies of ULD1 and ULD2

To assess the potential toxicities of ULD1 and ULD2, toxicology studies have been carried out. First, we have measured the viability of HepG2 human liver and MCF-7 human epithelial cell lines in the presence of ULD1 and ULD2, respectively, using a standard lactate dehydrogenase (LDH) assay. Neither ULD1 nor ULD2 was cytotoxic at concentrations up to the tested maximum, 100 μM (IC_50_ >100 μM, S8 Table). Next, to assess the potential genotoxicity of ULD1 and ULD2, we have tested their potential chromosomal aberration-causing effect on CHO-K1 hamster ovary cells, using a standard in vitro micronucleus assay [59]. No genetic toxicity of any of the 2 compounds have been detected (S9 Table). Potential cardiac safety and cross-reactivity to the human ether-a-go-go-related gene (hERG) potassium ion channel are also frequent issues for drugs targeting DNA gyrase and topoisomerase IV [9,12]. As required by European Medicine Agency (EMA) and the US Food and Drug Administration (FDA) [60, 61], we have tested both ULD1 and ULD2 for their inhibitory effects on the human hERG ion channels, at a concentration of 150 μM, using electrophysiological assays. No statistically significant inhibitory effect of either compound has been detected (S10 Table). We have also evaluated the hemolytic activity of ULD1 and ULD2 on human red blood cells, and again, no biocompatibility concerns have been raised (S11 Table).

### In vivo efficacy of ULD1 and ULD2

**Fig 4:**
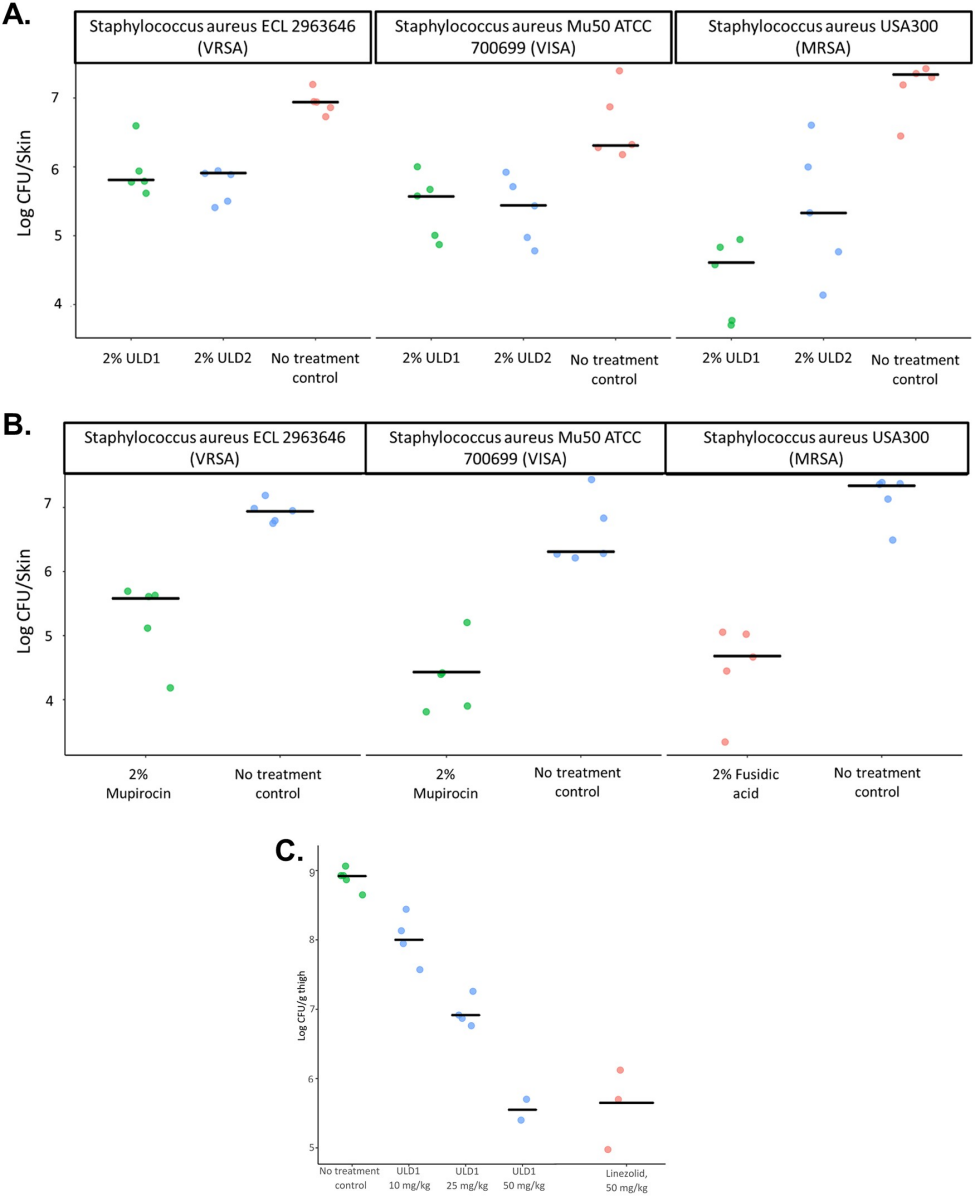
In vivo efficacy of ULD1 and ULD2 in 2 mouse infection models of *Staphylococcus aureus*. **A.** and **B.** In vivo efficacy of ULD1 and ULD2 in a mouse model of skin infection. Figure shows the number of CFUs after twice-daily topical antibiotic treatment (starting at 1 hour postinfection). Three different *S*. *aureus* strains were used as inoculum in 5 immunocompetent mice per each group. Fig A and B displays results with ULD1, ULD2 (**A**) and positive control antibiotics fusidic acid and mupirocin (**B**), respectively. Skin tissue CFUs were determined at 25 hours postinfection. CFUs from each mouse are plotted as dots; the black line represents the average CFU in each experimental group. **C.** Efficacy of ULD1 in a neutropenic mouse infection of *S*. *aureus*. The figure shows the number of CFUs in response to antibiotic treatment, using linezolid as positive control. A standard neutropenic thigh infection model was employed with *S*. *aureus* VISA ATCC 700699 (for details, see Materials and methods). CFUs from thigh tissue homogenates were determined at 26 hours postinfection. The CFUs from each individual are plotted as dots, black line represents the average CFU in each experimental group. The underlying data for these figures can be found in S2 Data. CFU, colony-forming unit; VISA, vancomycin-resistant *S*. *aureus*.

Based on the potent antibacterial activities of ULD1 and ULD2 and the lack of toxicity, we have finally tested their in vivo efficacy in mice models of *S*. *aureus* infections. First, a murine model of human staphylococcal SSTI has been utilized [62,63]. This preclinical model is extensively used to characterize the pharmacokinetic and pharmacodynamic properties of antistaphylococcal agents, as well as to predict their human clinical efficacy [53, 62–64]. Topical ULD1 and ULD2 treatments (in the form of ointments) were tested against *S*. *aureus* USA300 MRSA (BAA1556) and VISA and VRSA clinical isolates. USA300 MRSA clinical isolates are responsible for most community epidemics in the USA and are spreading worldwide [25]. Also, these 3 strains together are resistant to at least 9 distinct classes of antibiotics, including mupirocin, a last-resort antibiotic against SSTIs caused by multidrug-resistant *S*. *aureus* (S1 Data). Topical application of ULD1 and ULD2 has exerted a potent antibacterial activity (Fig 4A and 4B), comparable to that of mupirocin. Subsequent pharmacokinetic (PK) analyses indicate efficient skin penetration for both drugs, reaching a concentration of up to 300-times the MIC of ULD1 and ULD2 in wild-type *S*. *aureus* VISA (S12 Table).

Finally, we have tested ULD1 in a neutropenic model of murine thigh infection. Intravenous (IV) administration of the drug resulted in potent antibacterial activity against *S*. *aureus* VISA infection (Fig 4C). Notably, the antibacterial activity of ULD1 was comparable to that of linezolid, a widely used clinical agent against systemic MRSA infections [42], but resistance against this drug is emerging rapidly [20]. Taken together, these in vivo efficacy data indicate that the molecular scaffold underlying ULDs could serve as a basis for successful future therapeutic efforts against both topical and systemic *S*. *aureus* infections.

## Discussion

Antibiotic resistance frequently results from mutations within the target protein at amino acid positions that form direct interactions with the pharmaceutical agent [50,65,66]. To mitigate the spontaneous development of target-mediated resistance, we have rationally developed novel antibacterial compounds that simultaneously fulfill 2 criteria. First, they display a balanced dual-targeting activity against 2, essential bacterial targets, and second, they simultaneously establish interactions with multiple, evolutionary highly conserved amino acids of these target proteins. This new class of dual-targeting antibacterial compounds inhibit bacterial DNA gyrase and topoisomerase IV protein complexes and are structurally distinct from novobiocin, gepotidacin, and fluoroquinolone antibiotics. Two lead molecules of this series, ULD1 and ULD2, are potent inhibitors of the ATPase activities of GyrB and ParE. Importantly, we have achieved a superior and balanced enzyme inhibition of both target proteins compared with novobiocin, an inhibitor of GyrB that has reached clinical practice but was later withdrawn [67].

ULD1 and ULD2 exert broad-spectrum antibacterial activities against a wide range of pathogens, including multidrug-resistant clinical isolates. The efficacy of ULD1 and ULD2 was tested against a broad panel of *S*. *aureus* clinical strains, including recently isolated MRSA and VISA variants. Approved drugs with clinical relevance against staphylococcal infections fail to inhibit a significant fraction of these isolates, whereas both ULD1 and ULD2 are found to be potently active against all of them (MIC ≤ 1 μg/mL, see Fig 2). ULD1 and ULD2 are nonhemolytic, nongenotoxic, exert no cytotoxicity on multiple human cell lines, and have no inhibitory activity on hERG ion-channels. Furthermore, our data indicate that the compounds could be especially useful for the eradication of *S*. *aureus* in acidic environments, such as the human skin. Using murine models of multidrug-resistant staphylococcal skin and thigh infections, ULD1 was shown to display potent efficacy both via topical and systemic administration. These promising in vivo efficacy results, combined with a lack of toxicity and good skin penetration, indicate that the ULD series could be considered for the treatment of skin and skin-structure infections (SSSIs) such as acute bacterial SSSIs (ABSSSIs) and impetigo caused by multidrug resistant *S*. *aureus* [41,45].

ULD1 and ULD2 bypass existing and clinically widespread resistance mechanisms, including those that hinder the efficacy of other DNA gyrase and topoisomerase IV inhibitors. Additionally, de novo resistance mutations against these compounds are rare and have a limited impact on resistance level. The MPC (i.e., the concentration required to prevent the emergence of single-step mutants) is exceptionally low for both compounds. Remarkably, all isolated double mutants and laboratory evolved strains from long-term drug exposure have displayed low resistance level only, i.e., they could be inhibited by 1 μg/mL of ULD2. Thus, even combinations of specific resistance mutations provide only moderate changes in compound susceptibility. This is in sharp contrast to the results of a previous study on gepotidacin. Gepotidacin, an antibiotic candidate, selectively inhibits both bacterial DNA gyrase and topoisomerase IV [68], but a combination of 2 specific mutations in these target proteins provide as high as a 2,000-fold increment in resistance level (256 μg/mL) [8]. Multitarget antibiotics in theory should remain sensitive to other resistance mechanisms mediated by efflux pumps or by enzymatic inactivation. However, in our case, the laboratory evolution experiments further confirm that resistance by genomic mutations is exceedingly rare against ULD1/ULD2. Importantly, these findings do not exclude the possibility that resistance may eventually emerge through horizontal gene transfer from other species [56,57]. This issue should be investigated by functional metagenomic assays in the future [69].

Taken together, our data indicate ULD1 and ULD2 could serve as a basis for future therapeutic efforts against a range of gram-positive pathogens. Furthermore, these compounds inhibit gram-negative pathogens in vitro, albeit at higher concentations. Appropriate structural modifications could increase the potency of this structural-class against gram-negative pathogens as well. As these molecules are small (<500 Da), the addition of new moieties is feasible in future optimization efforts. Importantly, as ULD1 is manufactured from commercially available reagents in only 2 synthesis steps, upscaling to industrial-sized production should be relatively straightforward.

## Materials and methods

### Chemical design and synthesis of ULD1/ULD2

To improve the binding affinity of ULD0 to both target enzymes, we introduced 2 modifications, leading to ULD1. 4,5-dibromopyrrole was replaced by the 3,4-dichloro-5-methylpyrrole. In the terminal part of ULD0, the carboxylic acid group was found to be flexible, pointing toward the solvent without any direct contact with specific amino acid residues of the target proteins. Therefore, ULD1 was designed as a rigidified analog of ULD0 in which the aliphatic carboxylic acid group of ULD0 was replaced by an aromatic carboxylic acid. These modifications also gave a less acidic character of ULD1, a property that is beneficial for bacterial cell wall penetration. Next, we introduced a benzyloxy group at position 4 of the benzothiazole core, leading to ULD2. The purpose of this modification was to strengthen cation-π interaction with conserved Arg84/Arg77 by the introduction of this electron-donating group and to achieve additional hydrophobic interactions with conserved Pro87/Pro80 and other residues in the lipophilic floor of the binding site. Full details of the synthesis, purification, and characterization, including ^1^H NMR spectrum of all reported compounds, are provided in S3 Data. All reagents were obtained from commercial sources unless noted otherwise.

### Media and antibiotics

Cation-adjusted Mueller Hinton II Broth (MHBII) was used for the growth of bacteria under standard laboratory conditions, for antimicrobial susceptibility tests and for the selection of resistant variants. To prepare MHBII broth, 22 g of MHBII powder (Becton Dickinson and Co.) was dissolved in 1 L of water (3 g beef extract, 17.5 g acid hydrolysate of casein, and 1.5 g starch). For propagation and for antimicrobial susceptibility tests of fastidious bacteria, Brain-Heart-Infusion Broth (BHI) was used. To prepare BHI broth, 37 g of BHI powder (Carl Roth GmbH) was dissolved in 1 L of water (7.5 g pig brain infusion, 10 g pig heart infusion, 10 g peptone, 2 g glucose, 5 g NaCl, 2.5 g Na_2_HPO_4_). MHBII or BHI agar was prepared by the addition of 14 g Bacto agar (Molar Chemicals) to 1 L of broth. For frequency-of-resistance assays, 1% agarose-containing MHBII plates (Lonza, SeaKem LE agarose) were used to reduce drug-adsorption in media. All culture medium was sterilized by autoclaving for 15 minutes at 121 °C. Unless otherwise noted, antibiotics and chemicals were ordered from Sigma-Aldrich (vancomycin, novobiocin, fusidic acid), MedChemExpress (gepotidacin), Fluka Analytical (ciprofloxacin), and MedKoo Biosciences (delafloxacin).

### Antibiotic susceptibility measurements

MICs were determined using a standard serial broth microdilution technique according to the CLSI guidelines [70]. Briefly, bacterial strains were inoculated onto MHBII agar plates and were grown overnight at 37 °C. Next, 3 individual colonies from each strain were inoculated into 1 mL MHBII medium and propagated at 37 °C, 250 rpm overnight. In the cases of *Enterococcus* and *Streptococcus* sp., cells were plated to BHI agar plates, and BHI broth was used to determine MICs. To perform MIC assays, 12-step serial dilutions using a 2-fold dilution-steps of the given antibiotic (each dissolved in 100% DMSO) were generated in 96-well microtiter plates (Corning Inc). Following dilutions, each well was seeded with 5×10^4^ bacterial cells. All measurements were performed in 3 parallel replicates and to avoid possible edge effects in microwell plates, side rows (A and H) were filled with sterile medium. Following inoculations, plates were covered with lids and wrapped in polyethylene plastic bags to minimize evaporation but allow for O_2_ transfer. Plates were incubated at 37 °C under continuous shaking at 150 rpm for 18 hours. After incubation, OD_600_ of each well was measured using a BioTek Synergy 2 microplate reader. MIC was defined as the antibiotic concentration that inhibited the growth of the bacterial culture, i.e., the drug concentration at which the average OD_600_ increment of the 3 technical replicates was below 1.5-fold background OD increment. For pH-dependent MIC-determination, we relied on the same method with the modification that pH of MHBII was adjusted to 5.5 using 1 M HCl. Expanded panel antibacterial spectrum of ULD1, -2, and comparator antibiotics were tested at IHMA Europe Sàrl (Switzerland) and at Eurofins Pharmacology Discovery Services (Taiwan) in broth microdilution assays, according to the corresponding CLSI guidelines [70].

### Determination of inhibitory activities on *S*. *aureus* DNA gyrase and topoisomerase IV

Inhibitory activities of ULD1 and ULD2 on *S*. *aureus* DNA gyrase and *S*. *aureus* topoisomerase IV were determined by gel-based supercoiling assays (Inspiralis Ltd., Norwich, UK). In all experiments, the activity of the enzymes was determined and standardized prior to experimental analysis. In all cases, 1 unit (U) was defined as the amount of enzyme that is required to fully supercoil 1 μg of relaxed pBR322 plasmid DNA. All compounds were diluted in 100% (v/v) DMSO. Final assay concentration of DMSO was 5% (v/v). DNA gyrase (1 U, 6 nM final concentration) was incubated with 0.5 μg of relaxed pBR322 DNA in a 30 μL reaction at 37 °C for 30 minutes under the following conditions: 40 mM HEPES KOH (pH 7.6), 10 mM magnesium acetate, 10 mM DTT, 2 mM ATP, 500 mM potassium glutamate, and 0.05 mg/mL bovine serum albumin (BSA). Each reaction was stopped by the addition of 30 μL chloroform/iso-amyl alcohol (24:1) and 20 μL Stop Dye (40% sucrose (w/v), 100 mM Tris-HCl (pH 7.5), 10 mM EDTA, 0.5 μg/mL bromophenol blue), before being loaded on a 1.0% TAE gel and electrophorized at 80 V for 2 hours. Bands were then visualized by ethidium bromide staining and scanned (GeneGenius, Syngene, Cambridge, UK). Inhibition levels were measured by determining the relative fluorescence of the supercoiled band using GeneTools, Syngene, Cambridge (UK). All measurements were performed in quadruplicates.

### Time-dependent killing

Time-dependent killing experiments were performed as previously [53]. *S*. *aureus* ATCC 700699 VISA cells were inoculated to MHBII agar plates and grown overnight at 37 °C. Next, 3 independent stationer-phase starter cultures were grown at 37 °C, 250 rpm overnight, in 3 × 1 mL MHBII broth. Next, 100-fold diluted cultures were prepared from each in 20 mL fresh MHBII medium and grown at 37 °C, 250 rpm until early exponential phase (OD_600_ = 0.3–0.4; i.e., 5 × 10^5^ cells/mL). Bacteria were then challenged with antibiotics at 10× MIC concentrations of ULD1, ULD2, and comparator antibiotics novobiocin, fusidic acid, and vancomycin in MHBII broth. Cells were treated with antibiotics in Erlenmeyer flasks at 37 °C, 150 rpm for 48 hours. At different time intervals (3, 6, 12, 24, and 48 hours) 100 μL aliquots were removed, and 10-fold serial dilutions were prepared in MHBII medium. Next, 50 μL from each dilution were plated on MHBII agar plates, and plates were incubated overnight at 37 °C to calculate CFU per mL.

### Frequency-of-resistance assay

To determine the spontaneous frequency-of-resistance, approximately 10^10^ cells from stationary-phase MHBII broth cultures of *S*. *aureus* ATCC 700699 and *S*. *aureus* ATCC 43300 were plated to antibiotic-containing plates according to a standard protocol to determine frequency-of-resistance [49, 53]. Prior to plating, bacteria were grown overnight in MHBII medium at 37 °C, 250 rpm, collected by centrifugation, and washed once in equal volumes of MHBII broth. From this concentrated cell suspension (250 μL), approximately 10^10^ cells were then plated to each MHBII agarose plates. Using agarose instead of agar reduced drug-adsorption and improved the performance of the assay. Petri-dishes (145 mm) were filled with 40 mL MHBII agarose medium containing the selective drug at the desired concentration (i.e., 2×, 4×, 8×, 12×, and 16× MIC of each given antibiotic). All experiments were performed in at least 3 replicates. Plates were grown at 37 °C for 72 hours. Total CFUs were determined simultaneously in each experiment by plating appropriate dilutions to antibiotic-free MHBII agar plates. Finally, resistance frequencies for each strain were calculated by dividing the number of colonies formed after a 72-hour incubation at 37 °C by the initial viable cell count.

### Adaptive laboratory evolution

Adaptive laboratory evolution experiments followed an established protocol for automated laboratory evolution and aimed to maximize the drug-resistance increment during a fixed time period. At each transfer step, 10^7^ bacterial cells were transferred to a new culture and adaptation were performed by passaging 10 independent populations of *S*. *aureus* ATCC 700699 (VISA) strain in the presence of increasing ULD1, ULD2, and novobiocin concentrations. Experiments were conducted in 96-well plates, in MHBII medium, by utilizing a checkerboard layout to minimize and monitor cross-contamination. These 96-well deep-well plates (0.5 mL, polypropylene, V-bottom) were covered with sandwich covers (Enzyscreen BV) to ensure an optimal oxygen exchange rate and limit evaporation and were shaken at 150 rpm, 37 °C. Twenty μL of each evolving culture was parallelly transferred into 4 independent wells containing 350 μL fresh medium and an increasing concentration of tested drugs (i.e., 0.5×, 1×, 1.5×, and 2.5× the concentration of the previous concentration step). Following cell transfer, each culture was allowed to grow for 48 hours. At each transfer, cell growth was monitored by measuring the OD_600_ in a BioTek Synergy 2 plate reader. Only populations with (1) detectable growth (i.e., OD_600_ > 0.125) and (2) the highest drug concentration were selected for further transfer. Accordingly, only 1 of the 4 populations was retained for each independently evolving strain. This protocol was designed to avoid population extinction and to ensure that populations with the highest level of resistance are propagated further during adaptive evolution. Samples from each transfer were frozen at −80 °C after the addition of 15% DMSO as cryoprotectant. Adaptation of populations was terminated after 20 transfers, i.e., 40 days. Following adaptation, cells from each final population were spread onto MHBII agar plates, and individual colonies were isolated. Next, one colony from each adapted line were subjected to capillary sequencing of *gyrB* and *parE* to assess their genotype. Sequencing independently isolated colonies from the same plates demonstrated that colonies within the same adapted population were all isogenic.

### Capillary sequencing

Genotypes of the isolated clones from frequency-of-resistance assays as well as adaptive laboratory evolution experiment were checked by capillary sequencing. The drug-target regions of *gyrB* and *parE* genes were amplified by PCR using DreamTaq PCR 2X Master Mix (ThermoFisher Scientific): denaturation 95 °C, 3 minutes; 30 cycles: 95 °C, 30 seconds; 65 °C, 30 seconds; 72 °C, 1 minute; and final extension 72 °C, 3 minutes. The sequences of the corresponding PCR oligos are available in File S1 Data, 769B2F, SAGB1R were used to amplify *gyrB* from VISA strains and 769E2F, SAPE1R primers were used to amplify *parE*. PCR products were treated with ExoSAP-IT PCR Product Cleanup Reagent (ThermoFisher Scientific) for 15 seconds at 37 °C to hydrolyze excess primers and nucleotides. Samples were then subjected to capillary sequencing with the corresponding forward primer.

### Fitness measurements

We observed the growth phenotype of bacterial variants by assessing their growth at 37 °C in antibiotic-free BHI medium following established protocols [71]. To measure growth, we inoculated 5×104 cells from early stationary-phase cultures (prepared in MHBII medium) into 100 μL of BHI medium in a 96-well microtiter plate and monitored growth for approximately 48 hours. Bacterial growth was measured as the OD_600_ of cultures at any given time point. OD_600_ measurements were carried out every 5 minutes using BioTek Synergy 2 microplate reader while bacterial cultures were grown at 37 °C under continuous shaking. Each bacterial variant and their corresponding wild types were measured in 6 replicates. Finally, growth rates were calculated from the obtained growth curves according to a previously described procedure. Fitness was approximated by calculating the area under curve (AUC) [72]. AUC has been previously used as a proxy for fitness because it has the advantage to integrate multiple fitness parameters, such as the slope of exponential phase (i.e., growth rate) and the final biomass.

### SMRT sequencing-based analysis of target-mediated resistance

Pooled *S*. *aureus* clones, isolated from first-step frequency-of-resistance assays, were subjected long-read amplicon sequencing. First, bacterial colonies were picked up from 2 independent FoR libraries. Clones were inoculated in 100 μL MHBII medium in 96-well microtiter plates and were grown overnight at 37 °C. Stationer-phase cultures were mixed equally, and genomic DNA was isolated using GeneElute Bacterial Genomic DNA Kit (Sigma-Aldrich). Drug-target regions with flanking DNA regions were amplified by PCR using Q5 High-Fidelity 2X Master Mix (New England BioLabs): denaturation 98 °C, 3 minutes; 20 cycles: 98 °C, 15 seconds; 62 °C, 25 seconds; 72 °C, 1 minute 20 seconds; final extension 72 °C, 3 minutes, by using the barcoded amplification primers as described in S1 Data. Following PCRs, amplicons were purified using DNA Clean & Concentrator Kit (Zymo Research), eluted in water, and their DNA concentration was checked by using a Qubit 4 fluorimeter. Finally, samples were subjected SMRT sequencing on a Pacific Biosciences Sequel instrument using Sequel Polymerase v3.0, SMRT cells v3, and Sequencing chemistry v3.0 (Norwegian Sequencing Centre, UiO, Oslo, Norway). After sequencing, raw circular-consensus SMRT sequencing reads were demultiplexed according to their corresponding barcodes (see S1 Data) by using Demultiplex Barcodes pipeline on SMRT Link v5.1.0.26412 (SMRT Tools 4 v5.1.0.26366). A minimum barcode score of 26 was used to identify high-quality barcodes. Following demultiplexing, sequencing reads were mapped to their corresponding reference sequences (i.e., *gyrB* and *parE*) by using bowtie2 2.3.4 37 (http://bowtie-bio.sourceforge.net/bowtie2) in “very-sensitive” mode, and the nucleotide composition was extracted for each nucleotide position within the target regions. Finally, genotype-frequencies at each nucleotide position was quantified by measuring the distribution and ratio of nucleotide substitutions for each reference nucleotide position.

### Whole-genome sequencing of laboratory evolved lines

Following adaptive laboratory evolution of *S*. *aureus Mu50* ATCC 700699 under ULD2 stress, 6–6 random colonies (from spreading each 5 independently evolved lines on MHBII agar plates) were isolated and subsequently subjected to whole-genome sequencing on Illumina HiSeq 4000 sequencer. gDNA was isolated from each evolved line and the corresponding wild-type strain by using GeneElute (Sigma-Aldrich) gDNA isolation kit according to the manufacturer’s instructions. To perform DNA sequencing, sequencing libraries were constructed from the gDNAs by fragmenting samples to a mean fragment length of 300 bp. Next, sequencing libraries were prepared by using a TruSeq DNA PCR-Free Library Prep Kit (Illumina). Finally, sequencing libraries were sequenced on a single sequencing lane of Illumina HiSeq 4000 using a HiSeq 3000/4000 SBS Kit (300 cycles, FC-410-1003, Illumina) to generate 2 × 150 bp paired-end reads. To determine the variants and to annotate the mutations, we mapped sequencing reads to their corresponding reference genome (i.e., *S*. *aureus* subsp. aureus Mu50 DNA, complete genome 2,878,529 bp circular DNA BA000017.4) with the mem subcommand of bwa 0.7.12-r1039 (Burrows-Wheeler Aligner) [73]. The SNPs and INDELs were called with VarScan v2.3.9 [74] with the following settings: min-reads2 = 4, min-coverage = 30, min-var-freq = 0.1, min-freq-for-hom = 0.6, min-avg-qual = 20, strand-filter = 0. Only variants with prevalence higher than 60% were considered as genuine mutations. Following variant calling, mutations were also manually inspected within the aligned reads. Finally, the annotation of each mutation with genomic features was performed with the intersect subcommand of bedtools v2.25.0 [75].

### Multiple sequence alignments of GyrB and ParE

First, we downloaded the proteome of 1,108 phylogenetically diverse, human-associated bacterial strains with sequenced genomes and unique NCBI taxonomic identifier [76]. The list entails representative species belonging to Actinobacteria, Firmicutes, Bacteroidetes, Proteobacteria, Chlamydiae bacterial phyla. GyrB and ParE proteins were identified in each proteome by BLAST search implemented in DIAMOND (version v0.9.25.126) [77]. Subsequent multiple sequence alignments were carried out with MAFFT (version v7.271) [78]. Based on the co-crystal structures of GyrB and ParE (Protein Data Bank identifiers: 4uro and 4urn, respectively) with novobiocin, amino residues in the ATP-binding sites were identified using the PyMOL Molecular Graphics System, Version 2.3.2. Schrödinger, LLC. To visualize the phylogenetic conservation of each amino acid residue, sequence logos were computed with ggseqlogos R package [79].

### In silico binding mode analysis

First, the crystal structures of the ATPase domain-containing fragments of the GyrB and ParE subunits of DNA gyrase and topoisomerase IV of *S*. *aureus* were downloaded from the PDB Database (PDB ID: 3ttz and 4urn, respectively). In the design phase, ULD1 and ULD2 were docked in the ATP-binding site of *S*. *aureus* GyrB and ParE using Glide XP (extra precision) protocol as implemented in Schrödinger Software. Next, in the case of gyrase B, the highly flexible loop region (between residues 105–127, *S*. *aureus* GyrB numbering) was replaced based on the X-ray structure of the N-terminal 43-kDa fragment of the *E*. *coli* DNA gyrase subunit B [80] (PDB ID: 4wub). As for topoisomerase IV, the same loop region was reconstructed based on the protein sequence downloaded from UniProt (ID: P0C1S7) using the chimera model option of Schrödinger Software’s homology model building panel. We used the same pipeline to determine the binding mode of ULD1 and ULD2. The ULDs were docked into the model structures using Induced Fit Docking (IFD) protocol of Schrödinger Software without side-chain optimization. Next, the best 5 binding poses were selected as initial poses for 10 subsequent 10 ns long MD simulations to identify the stable binding poses of ULDs. The method is implemented in the Schrödinger Software using the ligand root-mean-square deviation (RMSD) as collective variable. In our investigations, the default settings of the software were used [81]. Furthermore, we also investigated the location of highly bound water molecules within the binding pocket, because of its previously hypothesized role on pyrrolamidobenzothiazoles’s binding [41], by using the trj_occupancy.py algorithm of Schrödinger [81].

### Protein purification

The gene encoding *S*. *aureus* GyrB (Uniprot: P0A0K8) was synthesized and cloned into a pET24a(+) vector with an N-terminal 6×His tag followed by a tobacco etch virus (TEV) protease cleavage site. The recombinant plasmid was transformed into *E*. *coli* BL21(DE3)R3 containing pRARE2 plasmid. A 2-L culture was grown in LB medium at 37 °C until an OD_600_ of about 0.6 and then moved to 18 °C. The culture was induced with IPTG at 0.5 mM and grown overnight. The cells were lysed by sonication and purified by immobilized metal affinity chromatography (IMAC). Fractions of interest were pooled and cleaved overnight with TEV protease followed by another IMAC to remove the 6×His tag and uncleaved protein material. The cleaved GyrB was further polished on a High Load 26/60 Superdex 200 PG (GE Healthcare) SEC column using 20 mM Tris, 0.1 M NaCl, 5% glycerol, 2 mM DTT (pH 8.0) as mobile phase. Fractions were analyzed on a reducing SDS-PAGE gel. The final sample had an estimated purity of >95% and a yield of 92 mg per liter of culture.

### Co-crystallization of ULD2 with *S*. *aureus* GyrB

*S*. *aureus* DNA GyrB24 at 10 mg/mL in 20 mM Tris (pH 8.0), 0.1 M NaCl, 5% glycerol, 2 mM DTT was co-crystallized with ULD2. *S*. *aureus* DNA GyrB24 was diluted to 1 mg/mL, and 1 mM ULD-2 was added to the protein. The protein was then concentrated to 10 mg/mL prior to crystallization. The crystals of *S*. *aureus* GyrB24 in complex with ULD2 formed successfully under the following conditions: 0.1 M imidazole/MES (pH 6.5), 0.06 M divalent cations (0.03 M magnesium chloride, 0.03 M calcium chloride), 37.5% (25% v/v MPD, 25% v/v PEG 1000, 25% w/v PEG 3350). Co-crystals with compound ULD2 were transferred to a cryo solution containing additional 25% MPD and then transferred to liquid nitrogen for cryo-cooling.

### X-ray data collection and structure refinement

A data set for compound ULD-2, bound to *S*. *aureus* GyrB, was collected at 100 K at station P11, DESY, Hamburg, Germany (λ = 1.03 Å) equipped with a Pilatus 6M detector. The data were processed using the software XDS [82] and Aimless [83] to 1.6 Å in space group C2. The structure was solved by molecular replacement in Phaser a previously published structure (PDB ID: 5CPH), with the compound removed as search model. The structure was refined to convergence using Refmac5 [84], and model building was carried out in Coot [85]. Restraints and coordinate files for the ligand were generated by the Jligand program [86]. TLS refinement was implemented in the final stages of the refinement and the structure was refined to an R and Rfree of 17.8% and 19.9%, respectively [87]. Statistics for the data set are shown in S13 Table.

### Haemolysis analysis

Human red blood cells (with a hemoglobin concentration of 150–160 g/L) were collected from healthy volunteers in EDTA blood-sampling tubes. Next, 600 μl of this EDTA-treated blood was washed in TBS buffer (10 mM TRIS, 150 mM NaCl [pH 7.2]) and centrifuged at 1,500 g for 1 minute until the supernatant became colorless. The aliquot of the final pellet (200 μL) was diluted to 5 mL with TBS buffer. ULD1 and ULD2 were dissolved in DMSO and diluted in TBS buffer, resulting a 200 μg/mL stock solution containing 10% DMSO. Next, 100 μL of red blood cell suspension was pipetted into sterile Eppendorf tubes together with 100 μL of 2-fold serial dilution of the compounds (final concentration ranged between 100 μg/mL–0.1 μg/mL in a final volume of 200 μL). The plate was incubated for one hour at 37 °C followed by centrifugation at 1,500 g for 1 minute to sediment all red blood cells. Next, all supernatants were subjected to LDH and Haemolysis-Icterus-Lipaemia (HIL) index determination on a Roche Modular P800 analyzer according to the manufacturer’s instructions. After completion of this step the rest of the supernatants were transferred to sterile 96-well plates for the measurement of their optical density at 560-nm wavelength (in a Multiskan FC microplate reader, Thermo Scientific). Melittin (Bachem) at a concentration of 50 μg/mL, and TBS and 10% DMSO/TBS were served as positive (100% hemolysis) and negative (no hemolysis) controls, respectively. Finally, the hemolytic effect of each compound at each concentration was calculated as follows:

$$({\text{Compound}\;\text{OD}}_{560\text{nm}}-{\text{TBS}\;\text{OD}}_{560\text{nm}})\times 100/({\text{Melittin}\;\text{OD}}_{560\text{nm}}-{\text{TBS}\;\text{OD}}_{560\text{nm}}).$$

### Mammalian cytotoxicity and genotoxicity measurements

Cytotoxicity of compounds on HepG2 and MCF-7 mammalian cell lines was determined by using a standard LDH assay. Briefly, HepG2 and MCF-7 cells were cultured in Eagle’s MEM medium (from Gibco) supplemented with 2 mM L-glutamine (Sigma), 100 U/mL penicillin (Sigma), and 100 μg/mL streptomycin (Sigma), and 10% FBS (Gibco) at 37 °C and under 5% CO_2_. Next, LDH assays were performed according to manufacturer instructions (Thermo Fisher Scientific, Massachusetts, USA). By using a CyQUANT LDH Cytotoxicity Assay Kit. Firstly, the cells were seeded in 96-well microtiter plates at 10^5^ cells/mL (100 μL/well) and allowed to attach overnight. Cells were then treated with selected compounds or with sterile ultrapure water (for the determination of spontaneous LDH activity) and incubated for 24 hours at 37 °C and under 5% CO_2_. Lysis buffer (10 μL, Thermo Fisher Scientific) was then added to the maximal LDH activity control wells and incubated additional 45 minutes. The cell culture supernatant (50 μL) was then transferred to a new 96-well plate and mixed with 50 μL of the reaction mixture. After 30 minutes on room temperature, reactions were stopped with 50 μL of the Stop solution. Absorbance (490 nm and 680 nm) was measured with the automatic microplate reader Synergy 4 Hybrid Microplate Reader (BioTek, VT, USA). All experiments were performed in triplicates and repeated 3 times. Statistical significance (*P* < 0.05) was calculated with 2-tailed Welch’s *t*-test between treated groups and DMSO. The percentage of cytotoxicity was determined according to the following equation:

$$\%Cytotoxicity=\left(\frac{Compound\;treated\;LDH\;activity-Spontaneous\;LDH\;activity}{Maximum\;LDH\;activity-Spontaneous\;LDH\;activity}\right)*100$$

Genetic toxicity analysis of compounds was performed in an in vitro micronucleus test, according to the protocol described in Diaz and colleagues (2007) [59], at Eurofins Panlabs (St. Charles, MO, US). Micronucleated cells and micronuclei were enumerated by the use of high-content fluorescent cell imaging. The assay was performed on CHO-K1 cells. Prior to imaging, cells were treated with ULD1 or ULD2 for 4 hours at 37 °C. Experiments were performed in 2 biological replicates. All cell lines were tested as being negative for *Mycoplasma* contamination.

### Human hERG Potassium ion-channel inhibition assay

Inhibition of the human hERG cardiac K+ ion channel was determined by Eurofins Panlabs (St. Charles, MO, USA) by using QPatch automated whole-cell patch-clamp electrophysiology, as described previously [88]. ULD1 and ULD2 were tested at 150 μM concentration. During measurements, after whole-cell patch configuration was achieved, the cells were held at −80 mV. Next, a 500-millisecond pulse to −40 mV was delivered to measure the leaking current, which was then subtracted from the tail current’s readout. Then the cell was depolarized to +40 mV for 500 milliseconds and then to −80 mV over a 100-millisecond ramp to elicit the hERG tail current. This workflow was repeated once in every 8 seconds to monitor the current amplitude. The parameters measured during each test were the maximum tail current evoked on stepping to 40 mV and ramping back to −80 mV from the test pulse. All data were filtered for seal quality, seal drop, and current amplitude. The peak current amplitude was calculated before and after compound addition, and the amount of inhibition was assessed by dividing the test compound’s current amplitude by the control’s current amplitude. During our tests, the control was the mean hERG current amplitude collected 15 seconds at the end of each measurement, and the test compound was the mean hERG current amplitude collected in the presence of test compound at the given concentration.

### *S*. *aureus* dermal infection model

The testing strains, *S*. *aureus* USA300 (MRSA) BAA1556, *S*. *aureus* (VRSA) ECL2963646, and *S*. *aureus* Mu50 (VISA) ATCC 700699 were obtained from frozen stocks and thawed at room temperature. Next, an aliquot of 0.2 mL from each stock was transferred to 20 mL BHI broth and incubated at 37 °C with shaking (120 rpm) for 8 hours. Cells in 20 mL culture were pelleted by centrifugation, 3,500*g* for 15 minutes, and then resuspended in 10 mL cold PBS, then cells were diluted in PBS to obtain the inoculum sizes of 1×10^5^ or 1× 10^6^ CFU/mL, based on the testing strain’s virulence. In all cases, the actual colony counts were determined by plating dilutions in at least 3 replicates onto MHBII agar plates followed by 24 hours’ incubation and colony counting.

For dermal infections, groups of 5 female ICR mice weighing 24 ± 4 g, provided by BioLasco Taiwan (under a Charles River Laboratories Technology Licensee), were used. Animals were acclimated for 3 days prior to use and were confirmed to be in good health. Prior to infection, animals were anesthetized with etomidate-lipuro emulsion (20 mg /10 mL) at 20 mg/kg by IV injection, and then the fur on the back was removed by an electric shaver, and the epidermal layer was disrupted with an abrasive paper according to the protocol developed by Kugelberg and colleagues [63]. Mice were inoculated topically on the wound area with testing strain suspension, 5 μL/mouse. The target inoculation densities were 1×10^5^ or 1× 10^6^ CFU/mL, based on the corresponding strain’s virulence. Following infection, animals were housed separately. Prior to treatment, ULD1 and −2 were dissolved in 100% DMSO and then further diluted in either corn oil (90% corn oil + 10% DMSO in the final ointment) or 0.5% water-based carboxymethyl cellulose (CMC) solution. Test articles were administered topically (20 μL/mouse) twice daily. One no-treatment group was euthanized at 1 hour after infection for initial bacterial counts, and the other treatment groups were dosed twice daily with the test compounds and sacrificed either at 25 hours or at 73 hours postinfection. Animals were euthanized with CO_2_ asphyxiation and the infected skin, a 2 cm^2^ area, was excised. The skin samples of the wound infection (around 2 cm^2^ areas) were then homogenized in 1 mL PBS (pH 7.4) with a polytron homogenizer. A 0.1 mL aliquot of each homogenate was used for serial 10-fold dilutions and plated onto MHBII agar plates for bacterial enumeration. Statistical significance (*P* <0.05) was assessed with 1-way ANOVA followed by Dunnett’s method. A significant (*P* <0.05) decrease in the bacterial counts of the treated animals compared with the vehicle control group was considered significant antimicrobial activity.

### Thigh infection model

Neutropenic mouse thigh infection experiments were performed on female CD-1 mice (Charles River Laboratories, USA). To induce neutropenia, mice received 2 doses of cyclophosphamide on days -4 and -1 with 150 mg/kg, and 100 mg/kg delivered intraperitoneally (IP), respectively. The inoculum of the testing strain, *S*. *aureus* Mu50 (VISA) ATCC 700699, was prepared from overnight agar plate cultures. To prepare bacterial inoculums, a portion of the plate was resuspended in sterile saline and adjusted to an OD of 0.12 at 625 nm. Next, the resulted bacterial suspension was further diluted to reach an infecting inoculum of 6.0×10^5^ CFU per each mouse. Mice were inoculated with 100 μL of the prepared bacterial suspension via intramuscular injection into the right rear thigh. Plate counts of the inoculum were also performed in each case to confirm inoculum concentration and the actual inoculum size was 6.9×10^5^ CFU/mouse. Prior to infection, ULD1 was formulated by dissolving the compound in 5% DMSO, 5% cremophor EL, and 50 mM potassium phosphate. Test agent was then dosed via IV administration at 2, 8, 14, and 20 hours postinfection. Beginning at 2 hours, postinfection mice were dosed with either test article or positive control antibiotic. Mice receiving test agents were dosed intravenously at 10 mL/kg. Linezolid was delivered as a subcutaneous dose. Four animals were dosed per group. One group of 4 mice were euthanized at initiation of therapy (T = 2 hours) and CFUs determined. All remaining mice were euthanized at 26 hours postinfection. At termination, thighs were aseptically excised, weighed, and homogenized to a uniform consistency in 2 mL of sterile saline. The homogenate was serially diluted and plated on bacterial growth media. The CFUs were enumerated after overnight incubation.

### Pharmacokinetics measurements

Pharmacokinetics measurement of ULD1 and ULD2 in skin tissue was conducted parallelly with the mice dermal infection study. In all cases, skin tissue samples were collected at the termination and homogenized following the same method as homogenate preparation for bacterial enumeration. Before measurement, protein precipitation was performed by treating the samples with acetonitrile. Analytics were the performed by liquid chromatography coupled mass spectrometry (LC/MS/MS) on an AB SCIEX QTRAP mass spectrometer in electrospray, positive ions ionization mode, and with Multiple Reaction Monitoring scan mode. Samples were analyzed on a Phenomenex Luna 5u Phenyl-Hexyl 50 × 2.0 mm HPLC column in a mobile phase of A: acetonitrile/formic acid = 100/0.2 (v/v), and mobile phase of B: water / formic acid = 100/0.2 (v/v). The column’s temperature was set to 30 °C, the injection volumes were 10 μL, and measurement time was 5 minutes in all cases. Oxybutynin (0.01 ng/μL in acetonitrile) was used as an internal standard for the measurement. Finally, the concentration of the drug in tissue samples was calculated from the peak area ratio.

### Ethics statement

Animal experiments for this study were performed by Eurofins Panlabs Taiwan Ltd. The Institutional Animal Care and Use Committee (IACUC) reviewed the planned experiments submitted under Protocol Number IM003-01132016 and provided official approval. The company also obtained an Animal Welfare Assurance (identification number A5890-01) from the Department of Health & Human Services.

## Supporting information

S1 Table Molecular modeling of drug–target interactions. *S*. *aureus* GyrB and ParE amino acid residues interacting with ULD1 and ULD2 as revealed by Induced Fit Docking and subsequent molecular dynamics simulations (See Materials and methods). GyrB, subunit B of DNA gyrase; ParE, subunit E of topoisomerase IV.
(XLSX)

S2 Table Interaction pattern of ULD1 with DNA GyrB and ParE, and the position of ULD1 resistance-conferring mutations. Table shows the position of first-step ULD1 resistance-conferring mutations within the drug’s binding pocket in GyrB and ParE at amino acid positions that are interacting with ULD1 (based on Induced Fit Docking and subsequent molecular dynamics simulation). Mutations have not been detected at interacting amino acids Asp81, Arg84, and Pro87 (in bold), in accordance with a prior study that has revealed that these residues are difficult to mutate as they have essential role in enzymatic function [39]. GyrB, subunit B of DNA gyrase; ParE, subunit E of topoisomerase IV.
(XLSX)

S3 Table MICs of ULD1 and ULD2 against ESKAPE pathogens and selected human pathogenic bacterial isolates. MIC measurements were performed in 3 replicates according to CLSI guidelines. -R, resistant; -NS, nonsensitive; CLSI, Clinical and Laboratory Standards Institute; GISA, glycopeptide-intermediate *S*. *aureus*; MIC, minimum inhibitory concentration; MSSA/VSSA, methicillin-sensitive/vancomycin-sensitive *S*. *aureus*; MRSA/VRSA, methicillin-resistant/vancomycin-resistant *S*. *aureus*; MRSE, methicillin-resistant *S*. *epidermidis*; VISA, vancomycin-intermediate *S*. *aureus*; VRE, vancomycin-resistant *Enterococcus*.
(XLSX)

S4 Table pH-dependence of antibiotic bioactivity against *S*. *aureus*. MIC measurements were determined by broth microdilution in 3 replicates according to CLSI guidelines. Star (*) denotes resistance according to FDA guidelines [89]. CLSI, Clinical and Laboratory Standards Institute; MIC, minimum inhibitory concentration.
(XLSX)

S5 Table Estimated frequency of missense mutations at the 4 most common, single-step mutational positions in GyrB against ULD1 in *Staphylococcus aureus* VISA ATCC 700699. Results are from 2 independent frequency-of-resistance assays followed by the Pacific Biosciences Single-Molecule Real-Time sequencing of *gyrB* and *parE* (see Materials and methods). GyrB, subunit B of DNA gyrase; ParE, subunit E of topoisomerase IV; VISA, vancomycin-intermediate *S*. *aureus*.
(XLSX)

S6 Table Resistance level of second-step spontaneous ULD1- and ULD2-resistant mutants in *S*. *aureus* ATCC 700699. MICs were determined in MHBII medium at 37 °C by broth microdilution assay according to CLSI guidelines. CLSI, Clinical and Laboratory Standards Institute; MHBII, Mueller Hinton II Broth; MIC, minimum inhibitory concentration.
(XLSX)

S7 Table Whole-genome sequencing-based mutational analysis of *S*. *aureus* VISA ATCC 700699 lines evolved under ULD2 stress. The table indicates mutations in evolved lines compared with the parental genome of *S*. *aureus* ATCC 700699 (GeneBank ID: BA000017.4). ATCC, American Type Culture Collection.
(XLSX)

S8 Table Cytotoxicity profiles of ULD1 and ULD2 in 2 different mammalian cell lines. Cell viabilities were measured after 1-day incubation of ULD1 and ULD2 in each cell line by quantifying LDH levels (see Materials and methods). The highest testing concentration was 100 μM. All tests were performed besides etoposide as a cytotoxic positive control. Etoposide IC_50_ = 20.1 ± 1.60 μM (HepG2 cells); IC_50_ = 34.9 ± 12.5 μM (MCF-7 cells). ± indicates SD based on 3 independent replicates. LDH, lactate dehydrogenase.
(XLSX)

S9 Table Genetic toxicity of pyrrolamidobenzothiazol compounds based on micronucleus test. Compounds were assayed after 4 hours’ treatment with ULD1 or ULD2 on CHO-K1 cells, using a standard protocol [59]. A negative test (-) result indicates *P* > 0.05 by *t*-test and percentage of micronucleated cells less than 2-fold higher than the background level.
(XLSX)

S10 Table Electrophysiological assays on human hERG potassium ion-channels. SD represents standard deviation based on *n* = 8. A reference hERG-inhibitor, E-4031 (Sigma-Aldrich) was included as a positive control and had a measured IC_50_ of 0.033 μM. Only results showing an inhibition higher than 50% are considered to represent significant effects of the tested compounds. hERG, human ether-a-go-go-related gene potassium ion channel.
(XLSX)

S11 Table Biocompatibility of ULD1 and ULD2, based on their hemolytic activity on human red blood cells. For details, see Materials and methods.
(XLSX)

S12 Table Concentrations of ULD1 and ULD2 in mouse skin tissue after the topical treatment of *S*. *aureus* ATCC 700699 infections. The concentrations of ULD1 and ULD2 were determined after 3 days of twice-a-day topical administration in mice (*n* = 3, 20 μL ointment/mouse, containing 2% ULD1 or ULD2, respectively).
(XLSX)

S13 Table Data collection and refinement statistics for compound ULD2 in complex with *S*. *aureus* GyrB (PDB entry 6TCK). PDB, Protein Data Bank.
(XLSX)

S1 Fig Interactions between ULD1 and ULD2 in the ATP-binding site of *S*. *aureus* GyrB and ParE. **A.** Diagram of interactions of ULD1 (left) and ULD2 (right) in the ATP-binding site of S. aureus GyrB and ParE. Hydrogen bonds are presented as black dashed lines, cation-π interactions as green dashed lines and a circle, and hydrophobic interactions by a green curve. Molecular dynamics simulations revealed that ULD1 and ULD2 form a hydrogen bond with Asp81/Asp74 (GyrB/ParE), cation-π interaction with Arg84/Arg77 (GyrB/ParE), and weak hydrophobic interaction with Pro87/Pro80 (GyrB/ParE), respectively. For the detailed interaction map of ULD1 and ULD2 with *S*. *aureus* GyrB and ParE, see S1 Table and S1 Fig. Figure was generated by PoseViewWeb. **B.** Co-crystal structure of *S*. *aureus* DNA gyrase subunit B (in gray cartoon, deposited to PDB as entry 6TCK) in complex with ULD2 (in cyan sticks). For clarity, only amino acids that are interacting with ULD2 are numbered and presented as sticks. Water molecule is presented as a red sphere, and hydrogen bonds are shown as dashed black lines. Pyrrolamide moiety of ULD2 forms a hydrogen bond between the pyrrole NH group and Asp81 side chain and a hydrogen bond between the amide carbonyl oxygen and a water molecule that is coordinated by Asp81 and Thr173. The pyrrole chlorine atoms and a methyl group are engaged in several hydrophobic interactions with Ile51, Val79, Ile102, Ile103, Thr173 and Ile175. Two additional hydrogen bonds are formed between the carboxylate of ULD2 and Arg144 side chain. The benzothiazole scaffold’s 4-benzyloxy group points to the lipophilic floor of the GyrB ATP-binding site, where it forms hydrophobic contacts with Pro87 and Ala98. GyrB, subunit B of DNA gyrase; ParE, subunit E of topoisomerase IV.
(TIF)

S2 Fig Time-dependent killing of *S*. *aureus* ATCC 700699 (VISA) by ULD1, ULD2, novobiocin, vancomycin, and fusidic acid. Bacterial cultures were grown to an early exponential phase and were subsequently diluted to 5×10^5^ cells/mL and challenged with antibiotics at 10× the wild-type MIC. The number of surviving cells were plotted as the function of time. The figure shows the average of 3 independent experiments. Error bars represent standard deviation. The underlying data for this figure can be found in S2 Data. MIC, minimum inhibitory concentration; VISA, vancomycin-intermediate *S*. *aureus*.
(TIF)

S3 Fig Resistance level of the single-step, spontaneous ULD1- and ULD2- resistant mutants of *S*. *aureus* VISA ATCC 700699. MICs were determined in MHBII medium at 37 °C by broth microdilution assay according to CLSI guidelines. The underlying data for this figure can be found in S2 Data. CLSI, Clinical and Laboratory Standards Institute; MHBII, Mueller Hinton II Broth; MIC, minimum inhibitory concentration; VISA, vancomycin-intermediate *S*. *aureus*.
(TIF)

S4 Fig Acquisition of second-step ULD1 and ULD2 resistance-conferring mutations in A) *S*. *aureus* VISA ATCC 700699 GyrB R144I and B) *S*. *aureus* VISA ATCC 700699 GyrB I175T. Data are based on 3 independent biological replicates. Error bars indicate standard error. Double asterisks (**) mark samples with a frequency-of-resistance of >1×10^−6^. The underlying data for these figures can be found in S2 Data. VISA, vancomycin-intermediate *S*. *aureus*.
(TIF)

S5 Fig Fitness (A) and growth phenotype (B) of ULD2-evolved and wild-type *S*. *aureus* VISA ATCC 700699. Fitness was approximated from the growth curves of isogenic microbial populations (see Materials and methods). Measurements were performed in 6 replicates. The underlying data for this figure can be found in S2 Data. Growth phenotypes were observed in BHI agar plates and documented after 24 hours of incubation at 37 °C. BHI, Brain-Heart-Infusion Broth; VISA, vancomycin-intermediate *S*. *aureus*.
(TIF)

S1 Data Oligonucleotide list and detailed results of MIC measurements. MIC, minimum inhibitory concentration.
(XLSX)

S2 Data Raw data for the figures and supporting figures featured in the manuscript. (XLSX)

S3 Data Details of the synthetic chemistry. (PDF)

## Abbreviations

ABSSSI: acute bacterial skin and skin-structure infection
EMA: European Medicine Agency
ESKAPE pathogens: *Staphylococcus aureus*, *Enterococcus* sp., *Pseudomonas aeruginosa*, *Klebsiella pneumoniae*, *Acinetobacter baumannii*
FDA: US Food and Drug Administration
GyrB: subunit B of DNA gyrase
hERG: human ether-a-go-go-related gene potassium ion channel
IV: intravenous
LDH: lactate dehydrogenase
MIC: minimum inhibitory concentration
MPC: mutant prevention concentration
MRSA: methicillin-resistant *Staphylococcus aureus*
OD_600_: optical density at 600 nm
ParE: subunit E of topoisomerase IV
PK: pharmacokinetic
SMRT: single-molecule real-time
SSSI: skin and skin-structure infection
SSTI: skin and soft-tissue infections
UDP: uridine diphosphate
VISA: vancomycin-intermediate *Staphylococcus aureus*
VRE: vancomycin-resistant Enterococcus
