# Supplementary figures and images for: Rational design of balanced dual-targeting antibiotics with limited resistance
Source: PLoS Biol. 2020 Oct 5;18(10):e3000819. doi: 10.1371/journal.pbio.3000819 (PMC7561186; doi:10.1371/journal.pbio.3000819)

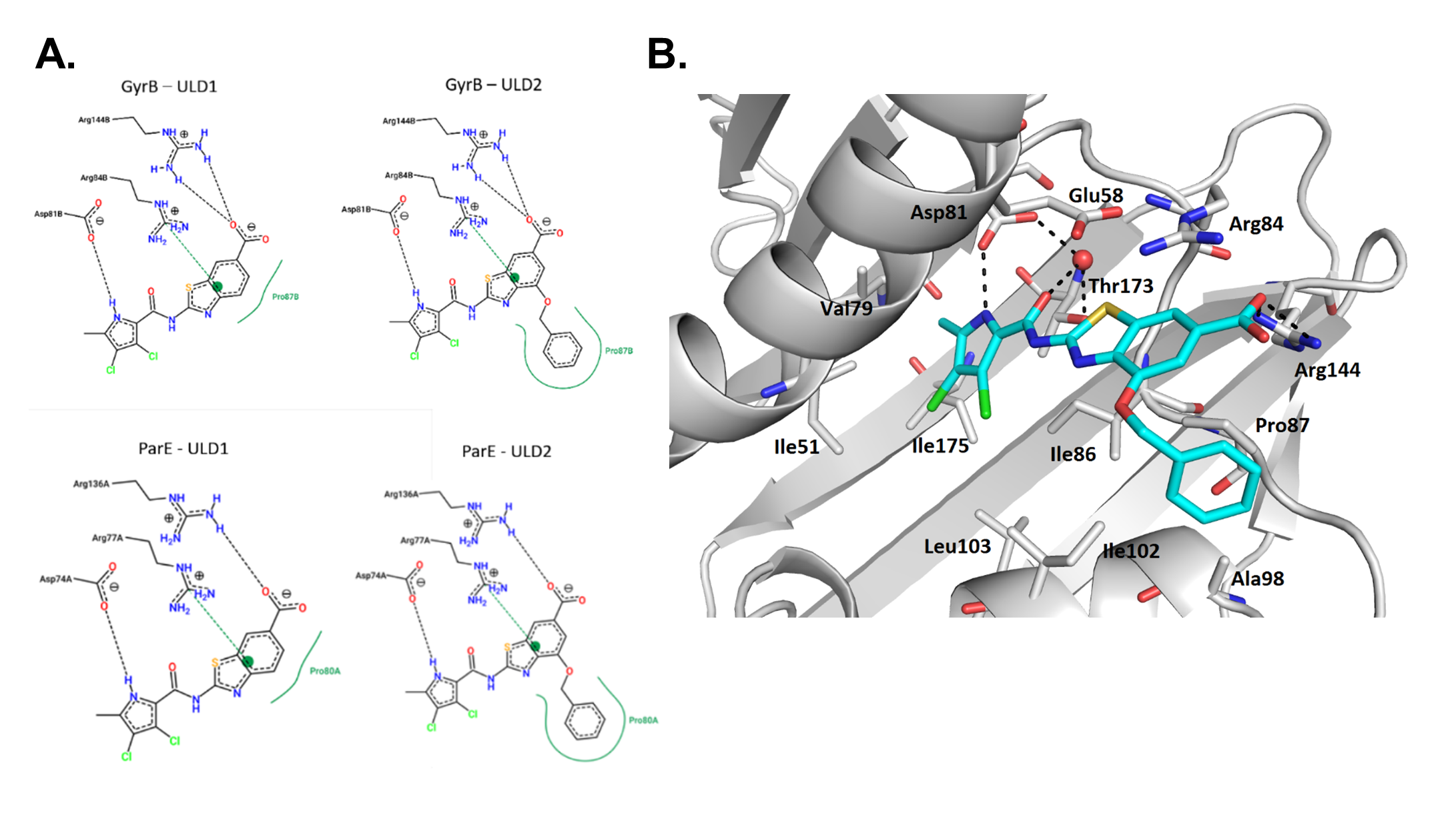

Supplement: S1 Fig — A. Diagram of interactions of ULD1 (left) and ULD2 (right) in the ATP-binding site of S. aureus GyrB and ParE. Hydrogen bonds are presented as black dashed lines, cation-π interactions as green dashed lines and a circle, and hydrophobic interactions by a green curve. Molecular dynamics simulations revealed that ULD1 and ULD2 form a hydrogen bond with Asp81/Asp74 (GyrB/ParE), cation-π interaction with Arg84/Arg77 (GyrB/ParE), and weak hydrophobic interaction with Pro87/Pro80 (GyrB/ParE), respectively. For the detailed interaction map of ULD1 and ULD2 with S. aureus GyrB and ParE, see S1 Table and S1 Fig. Figure was generated by PoseViewWeb. B. Co-crystal structure of S. aureus DNA gyrase subunit B (in gray cartoon, deposited to PDB as entry 6TCK) in complex with ULD2 (in cyan sticks). For clarity, only amino acids that are interacting with ULD2 are numbered and presented as sticks. Water molecule is presented as a red sphere, and hydrogen bonds are shown as dashed black lines. Pyrrolamide moiety of ULD2 forms a hydrogen bond between the pyrrole NH group and Asp81 side chain and a hydrogen bond between the amide carbonyl oxygen and a water molecule that is coordinated by Asp81 and Thr173. The pyrrole chlorine atoms and a methyl group are engaged in several hydrophobic interactions with Ile51, Val79, Ile102, Ile103, Thr173 and Ile175. Two additional hydrogen bonds are formed between the carboxylate of ULD2 and Arg144 side chain. The benzothiazole scaffold’s 4-benzyloxy group points to the lipophilic floor of the GyrB ATP-binding site, where it forms hydrophobic contacts with Pro87 and Ala98. GyrB, subunit B of DNA gyrase; ParE, subunit E of topoisomerase IV. (TIF) [file pbio.3000819.s014.tif]

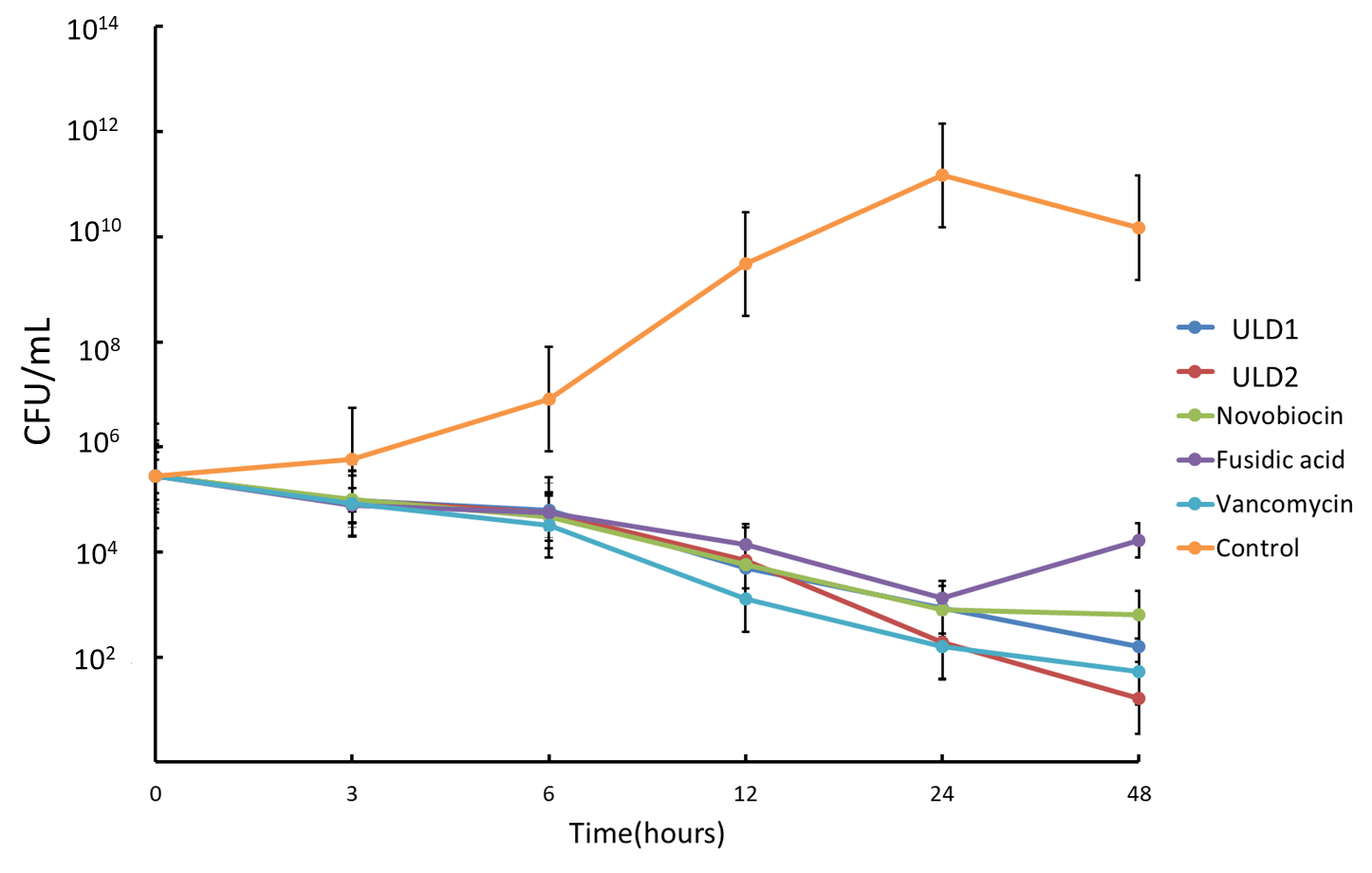

Supplement: S2 Fig — Bacterial cultures were grown to an early exponential phase and were subsequently diluted to 5×10^5^ cells/mL and challenged with antibiotics at 10× the wild-type MIC. The number of surviving cells were plotted as the function of time. The figure shows the average of 3 independent experiments. Error bars represent standard deviation. The underlying data for this figure can be found in S2 Data. MIC, minimum inhibitory concentration; VISA, vancomycin-intermediate S. aureus. (TIF) [file pbio.3000819.s015.tif]

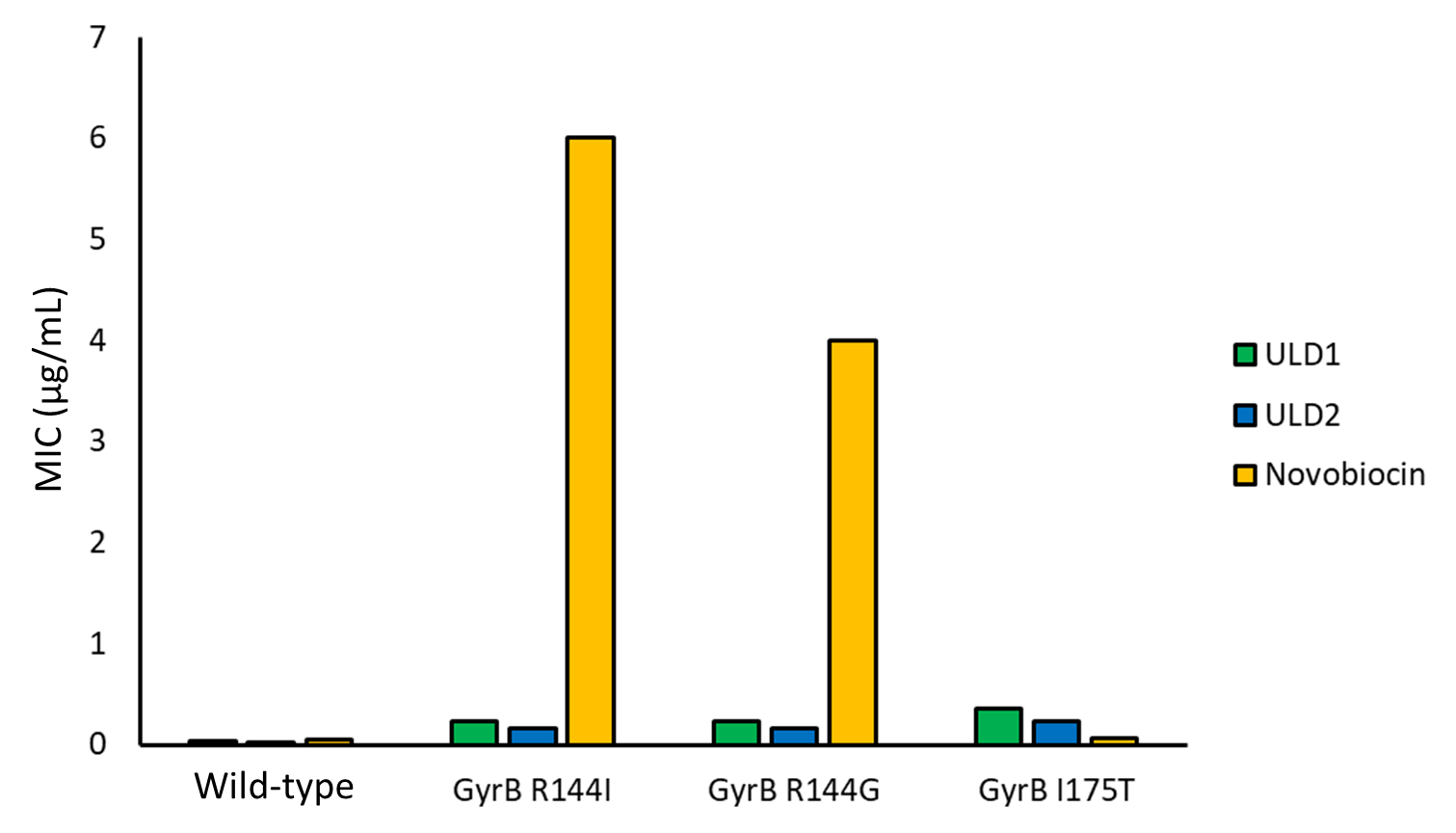

Supplement: S3 Fig — MICs were determined in MHBII medium at 37 °C by broth microdilution assay according to CLSI guidelines. The underlying data for this figure can be found in S2 Data. CLSI, Clinical and Laboratory Standards Institute; MHBII, Mueller Hinton II Broth; MIC, minimum inhibitory concentration; VISA, vancomycin-intermediate S. aureus. (TIF) [file pbio.3000819.s016.tif]

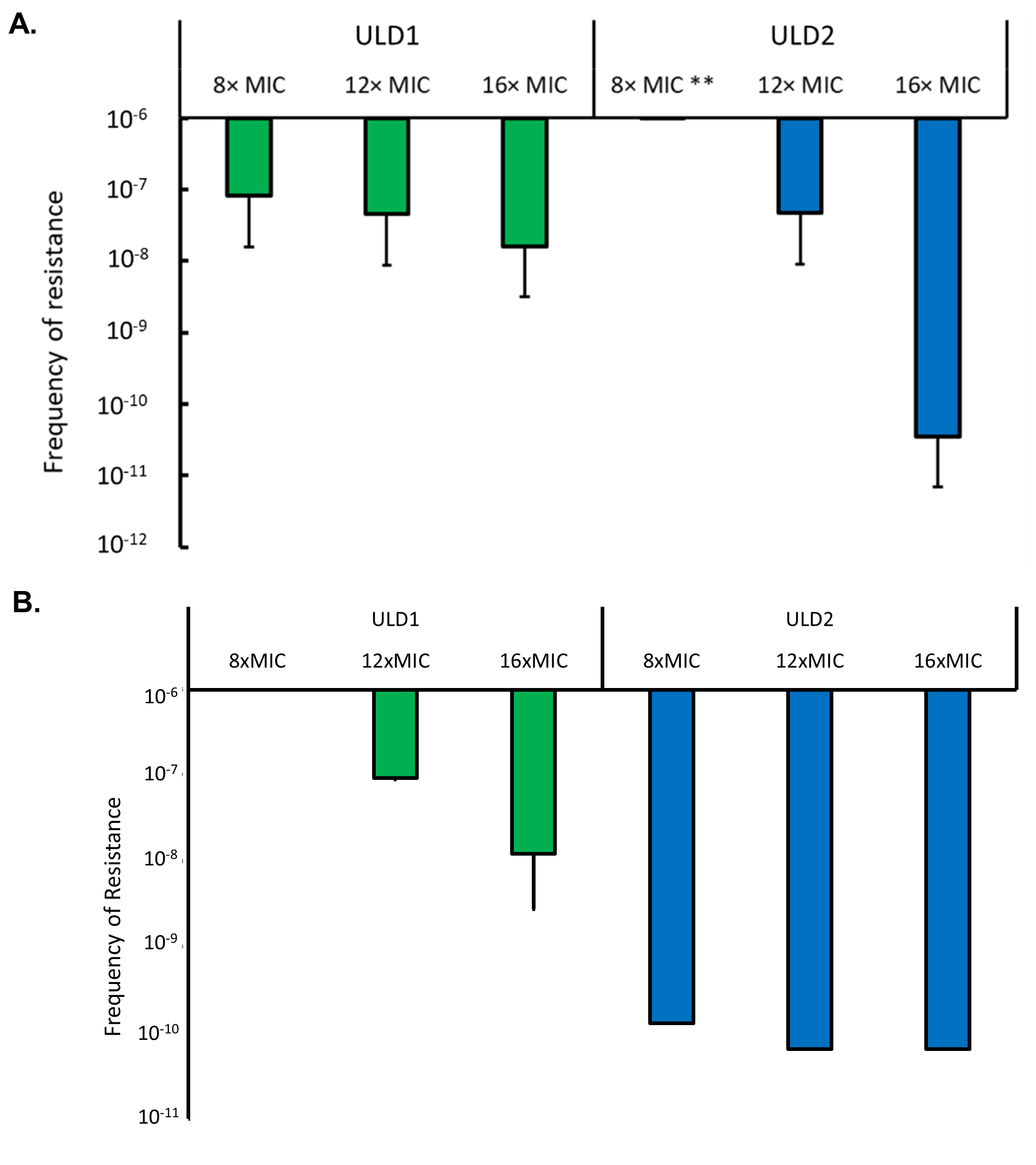

Supplement: S4 Fig — Data are based on 3 independent biological replicates. Error bars indicate standard error. Double asterisks (**) mark samples with a frequency-of-resistance of >1×10^−6^. The underlying data for these figures can be found in S2 Data. VISA, vancomycin-intermediate S. aureus. (TIF) [file pbio.3000819.s017.tif]

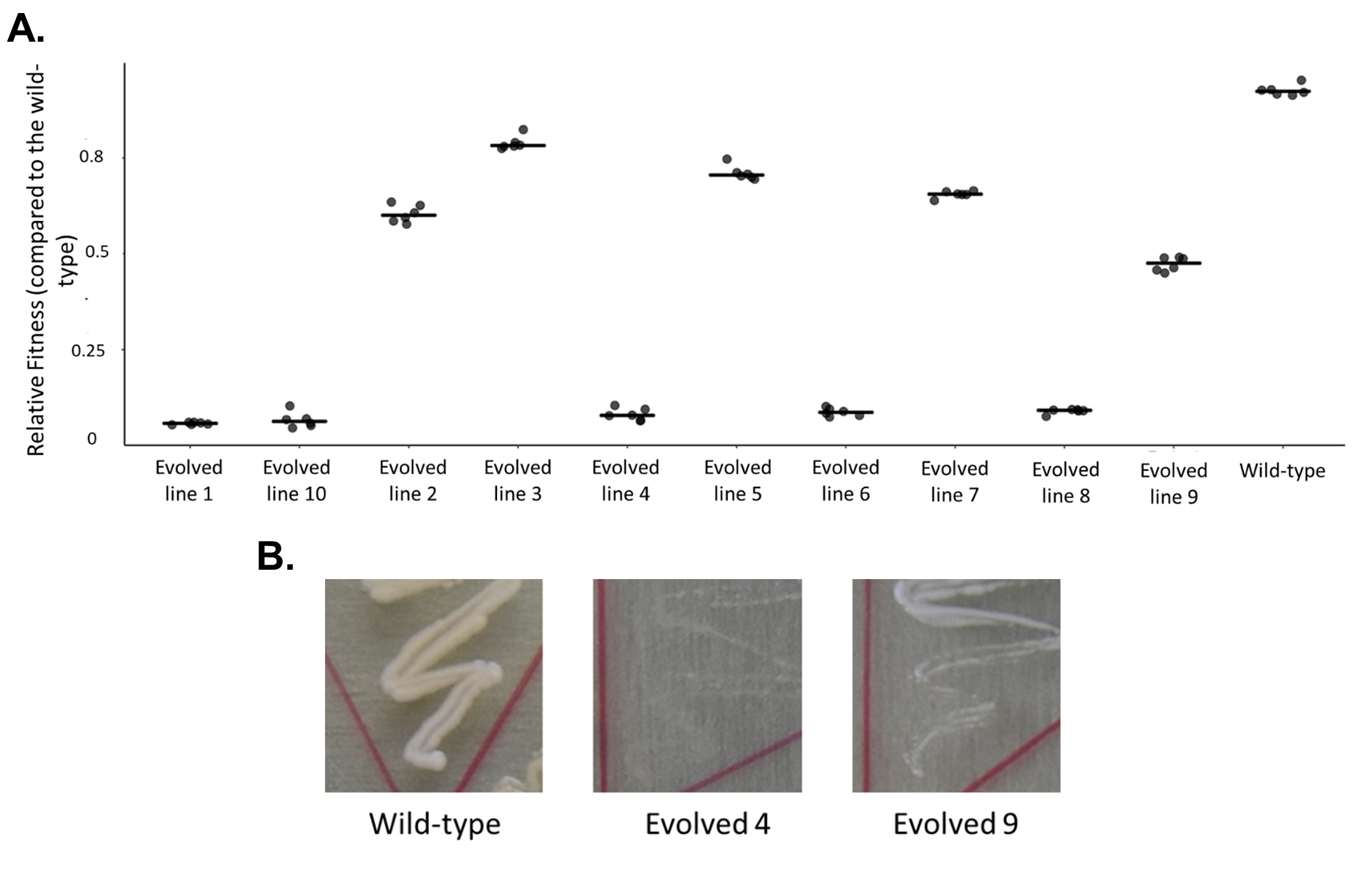

Supplement: S5 Fig — Fitness was approximated from the growth curves of isogenic microbial populations (see Materials and methods). Measurements were performed in 6 replicates. The underlying data for this figure can be found in S2 Data. Growth phenotypes were observed in BHI agar plates and documented after 24 hours of incubation at 37 °C. BHI, Brain-Heart-Infusion Broth; VISA, vancomycin-intermediate S. aureus. (TIF) [file pbio.3000819.s018.tif]
