# Supplementary material for: Rational design of balanced dual-targeting antibiotics with limited resistance
Source: PLoS Biol. 2020 Oct 5;18(10):e3000819. doi: 10.1371/journal.pbio.3000819 (PMC7561186; doi:10.1371/journal.pbio.3000819)
Supplement: S3 Data — (PDF) [file pbio.3000819.s021.pdf]

Supporting Note I.

Synthetic chemistry:

Chemicals were obtained from Acros Organics (Geel, Belgium), Sigma-Aldrich (St. Louis, MO, USA) and Apollo Scientific (Stockport, UK) and used without further purification. Analytical TLC was performed on silica gel Merck 60 F_{254} plates (0.25 mm), using visualization with UV light and spray reagents. Column chromatography was carried out on silica gel 60 (particle size 240–400 mesh). HPLC analyses were performed on a Thermo Scientific Dionex Ultimate 3000 Binary Rapid Separation LC System (Thermo Fisher Scientific, Waltham, MA, USA) with an autosampler, a binary pump system, a photodiode array detector, a thermostated column compartment, and a Chromeleon Chromatography Data System. The column used was Agilent Eclipse C18 column (5 µm, 4.6 × 150 mm). The eluent consisted of trifluoroacetic acid (0.1% in water) as solvent A and acetonitrile as solvent B. Method was 30-90% of acetonitrile in TFA (0.1%) in 16 min, 90% acetonitrile to 20 min, a flow rate of 1.0 mL/min and a sample injection volume of 5 µL or 10 µL. ^{1}H and ^{13}C NMR spectra were recorded at 400 and 101 MHz, respectively, on a Bruker AVANCE III 400 spectrometer (Bruker Corporation, Billerica, MA, USA) in DMSO-d_6 or $CDCl_3$ solutions, with TMS as the internal standard. Mass spectra were obtained using Exactive Plus Orbitrap mass spectrometer (Thermo Fisher Scientific, Waltham, MA, USA). The purity of the tested compounds was ≥ 95% as established by HPLC.

Synthesis:

Synthesis of **ULD1** is presented in Supporting Scheme 1. To prepare compound **3** a two-step coupling reaction was performed between 3,4-dichloro-5-methyl-1*H*-pyrrole-2-carboxylic acid (**1**) and commercially available methyl 2-aminobenzo[*d*]thiazole-6-carboxylate (**2**). Using oxalyl chloride, **1** was first converted to acid chloride which was in the second step reacted with **2** at 130 °C in toluene. The obtained compound **3** was submitted to alkaline hydrolysis using 2 M NaOH to obtain final compound **4** (**ULD1**).

Synthesis of **ULD2** is shown in Supporting Scheme 2. First, 3-hydroxy-4-nitrobenzoic acid (**5**) was converted to methyl ester (**6**) using H_2SO_4 in methanol. Compound **6** was alkylated with benzyl group using benzyl bromide and K_2CO_3 to get **7** and in the next step nitro group of **7** was reduced to amino (**8**) in the presence of $SnCl_2$. Cyclisation of compound **8** with KSCN and bromine yielded benzothiazole **9** which was coupled to 3,4-dichloro-5-methyl-1*H*-pyrrole-2-carboxylic acid (**1**) in the two-step coupling reaction using oxalyl chloride in the first step and toluene in the second step. Finally, methyl ester of the obtained compound **10** was hydrolysed with 1 M NaOH to the final carboxylic acid **11** (**ULD2**).

Supporting Scheme 1: Synthesis of ULD1[a]

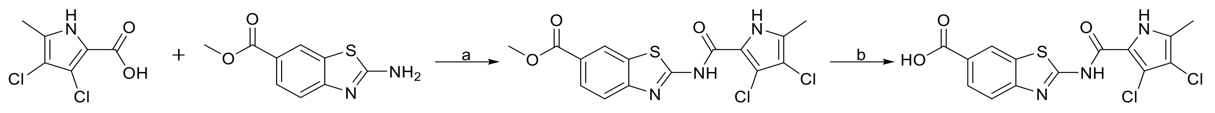

1 **2** **3** **4 (ULD1)**

[a]Reagents and conditions: (a) *i*) **1**, oxalyl chloride, CH_2Cl_2, rt, 15 h, then *ii*) **2**, toluene, 130 °C, 15 h; (b) 2 M NaOH, MeOH, 80 °C, 48 h.

Supporting Scheme 2: Synthesis of ULD2[a]

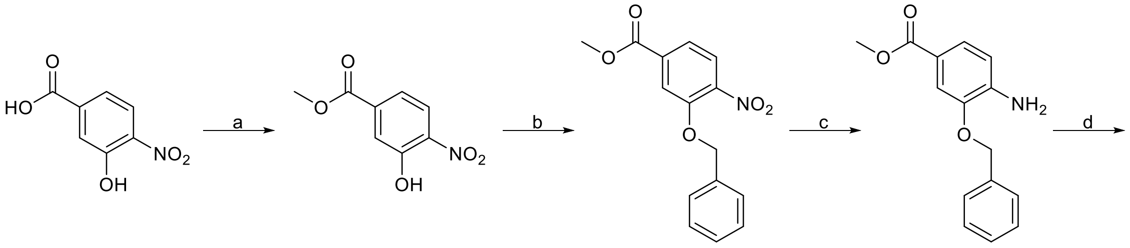

5 **6** **7** **8**

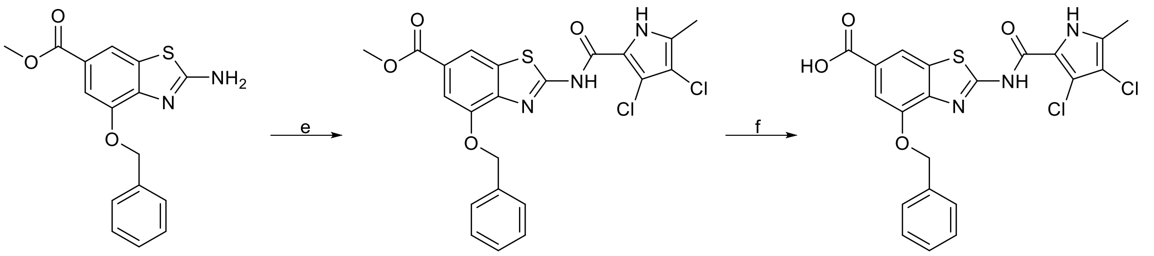

9 **10** **11 (ULD2)**

[a]Reagents and conditions: (a) H_2SO_4, MeOH, 65 °C, 15 h; (b) benzyl bromide, CH_3CN, 70 °C, 15 h; (c) $SnCl_2$, MeOH/EtOAc, 55 °C, 15 h; (d) KSCN, Br_2, CH_3COOH, 10 °C to rt, 15 h; (e) *i*) **1**, oxalyl chloride, CH_2Cl_2, rt, 15 h, then *ii*) **9**, toluene, 130 °C, 15 h; (f) 1 M NaOH, MeOH, 40 °C, 48 h.

Synthetic Procedures:

Methyl 2-(3,4-dichloro-5-methyl-1*H*-pyrrole-2-carboxamido)benzo[*d*]thiazole-6-carboxylate (3). To a suspension of 3,4-dichloro-5-methyl-1*H*-pyrrole-2-carboxylic acid (**1**, 244 mg, 1.26 mmol) in anhydrous dichloromethane (15 mL) oxalyl chloride (0.539 mL, 6.29 mmol) was added dropwise and the solution stirred at room temperature under argon atmosphere overnight. The solvent was evaporated under reduced pressure, methyl 2-aminobenzo[*d*]thiazole-6-carboxylate (**2**, 262 mg, 1.26 mmol) and toluene (20 mL) were added and the suspension was stirred at 130 °C overnight. The precipitate in the reaction mixture was filtered off, suspended in 1 M HCl (100 mL), sonicated and filtered off. The crude product was dispersed in methanol (100 mL), heated, filtered off and dried. Yield: 373 mg (77.2%); grey solid. ^1H NMR (400 MHz, DMSO-d_6) δ 2.29 (s, 3H), 3.89 (s, 3H), 7.83 (s, 1H), 8.04 (dd, *J* = 8.5, 1.8 Hz, 1H), 8.67 (s, 1H), 11.98 (s, 1H), 12.35 (s, 1H). HRMS (ESI^+) m/z for C15H12Cl2N3O3S ($[M+H]^+$): calculated 383.9971, found 383.9966.

2-(3,4-Dichloro-5-methyl-1*H*-pyrrole-2-carboxamido)benzo[*d*]thiazole-6-carboxylic acid (4 (ULD1)). Methyl 2-(3,4-dichloro-5-methyl-1*H*-pyrrole-2-carboxamido)benzo[*d*]thiazole-6-carboxylate (**3**, 389 mg, 1.01 mmol) was suspended in methanol (20 mL), 2 M NaOH (2.53 mL, 5.06 mmol) was added and the reaction mixture was stirred at 80 °C overnight. 500 µL of 2 M NaOH was added and the reaction mixture was stirred at 80 °C overnight. The solvent was removed under reduced pressure, the residue was acidified with 1 M HCl to pH = 1 and the obtained precipitate was filtered off. The crude product was purified with flash column chromatography using ethyl acetate/methanol (20/1) as an eluent. Yield: 189 mg (50.5%); white solid. ^{1}H NMR (400 MHz, DMSO-d_6) δ 2.28 (s, 3H), 7.79 (d, J = 8.5 Hz, 1H), 8.02 (dd, J = 8.5, 1.7 Hz, 1H), 8.62 (d, J = 1.4 Hz, 1H), 11.42-12.40 (m, 1H), 12.48 (s, 1H) (S1 Fig). ^{13}C NMR (101 MHz, DMSO-d_6) δ 10.95, 109.90, 115.55, 117.14, 119.36, 123.84, 125.73, 127.41, 129.93, 131.24, 150.68, 157.58, 161.58, 167.00 (S2 Fig). HRMS (ESI$^-$) *m/z* for $C_{14}H_8Cl_2N_3O_3S$ ([M-H]$^-$): calculated 367.9658, found 367.9671. HPLC: t_r 8.030 min (99.4 % at 254 nm) (S3 Fig).

Methyl 3-hydroxy-4-nitrobenzoate (6). To a suspension of 3-hydroxy-4-nitrobenzoic acid (**5**, 10.0 g, 54.6 mmol) in methanol (200 mL) cooled on ice bath thionyl chloride (11.9 mL, 163.8 mmol) was added dropwise. The mixture was stirred at 65 °C overnight upon which a clear solution formed. The solvent was evaporated under reduced pressure. The residue was dissolved in ethyl acetate (150 mL) and the organic phase was washed with water (60 mL), saturated aqueous $NaHCO_3$ solution (32 × 60 mL) and brine (2 × 50 mL), dried over Na_2SO_4, filtered and the solvent removed *in vacuo*. Yield: 9.64 g (89.6%); yellow solid. ^{1}H NMR (400 MHz, $CDCl_3$) δ 3.97 (s, 3H), 7.62 (dd, J = 8.8, 1.8 Hz, 1H), 7.84 (d, J = 1.7 Hz, 1H), 8.18 (d, J = 8.8 Hz, 1H), 10.51 (s, 1H).

Methyl 3-(benzyloxy)-4-nitrobenzoate (7). To a suspension of methyl 3-hydroxy-4-nitrobenzoate (**6**, 1.19 g, 6.02 mmol) and K_2CO_3 (1.66 g, 12.04 mmol) in acetonitrile (20 mL) benzyl bromide (0.788 mL, 6.62 mmol) was added dropwise and the reaction mixture was stirred at 60 °C overnight. The solvent was removed *in vacuo* and the residue dissolved in ethyl acetate (30 mL) and washed with water (2 × 20 mL) and brine (20 mL). The organic phase was dried over Na_2SO_4, filtered and the solvent evaporated under reduced pressure. Yield: 1.54 g (89.6%); yellow crystals. ^{1}H NMR (400 MHz, DMSO-d_6) δ 3.92 (s, 3H), 5.41 (s, 2H), 7.29-7.51 (m, 5H), 7.70 (dd, J = 8.3, 1.6 Hz, 1H), 7.90 (d, J = 1.5 Hz, 1H), 8.03 (d, J = 8.3 Hz, 1H).

Methyl 4-amino-3-(benzyloxy)benzoate (8). To a solution of methyl 3-(benzyloxy)-4-nitrobenzoate (**7**, 1.48 g, 5.17 mmol) in ethyl acetate/methanol (1.5:1, 25 mL), $SnCl_2$ (4.90 g, 25.8 mmol) was added and the reaction mixture stirred at 55 °C overnight. The solvent was removed *in vacuo* and to the residue $NaHCO_3$ (220 mL) was added dropwise on an ice bath. The obtained white suspension was sonicated for 30 min. Ethyl acetate was added and the precipitate was filtered off. The phases in the mother

liquid were separated and water phase was extracted with additional ethyl acetate. The precipitate was also resuspended in ethyl acetate and filtered again. The combined organic phases were washed with brine, dried over Na_2SO_4, filtered and the solvent removed *in vacuo*. Yield: 1.23 g (92.6%); dark yellow solid. ^{1}H NMR (400 MHz, DMSO-d_6) δ 3.75 (s, 3H), 5.17 (s, 2H), 5.68 (br s, 2H), 6.68 (d, 1H), 7.29-7.45 (m, 5H), 7.49-7.56 (m, 2H).

Methyl 2-amino-4-(benzyloxy)benzo[*d*]thiazole-6-carboxylate (9). To the solution of 4-amino-3-(benzyloxy)benzoate (**8**, 229 mg, 0.89 mmol) in acetic acid (4 mL) KSCN (346 mg, 3.56 mmol) was added and the solution stirred at rt for 20 min. The reaction mixture was cooled to 10 °C and bromine (0.092 mL, 1.78 mmol) in acetic acid was added dropwise upon which the solution turned to yellow suspension. The reaction mixture was then stirred at room temperature overnight. The reaction mixture was neutralized with 25% aqueous NH_3 solution (mL) to pH = 8 and the precipitate filtered off. To the crude product methanol (100 mL) was added, the mixture was heated to reflux, the undissolved solid was filtered off and washed successively with hot methanol. Methanol was removed under reduced pressure to obtain product as orange solid. Yield: 250 mg (89.3%); orange solid. ^{1}H NMR (400 MHz, DMSO-d_6) δ 3.83 (s, 3H), 5.24 (s, 2H), 7.32-7.44 (m, 3H), 7.46 (d, *J* = 1.6 Hz, 1H), 7.48-7.53 (m, 2H), 7.91 (s, 2H), 7.98 (d, *J* = 1.5 Hz, 1H). ^{13}C NMR (101 MHz, DMSO-d_6) δ 51.93, 69.96, 109.90, 115.89, 122.18, 127.85, 128.34, 131.80, 136.95, 146.64, 147.96, 166.08, 168.70.

Methyl 4-(benzyloxy)-2-(3,4-dichloro-5-methyl-1*H*-pyrrole-2-carboxamido)benzo[*d*]thiazole-6-carboxylate (10). To a suspension of 3,4-dichloro-5-methyl-1*H*-pyrrole-2-carboxylic acid (**1**, 167 mg, 0.859 mmol) in anhydrous dichloromethane (10 mL) oxalyl chloride (0.368 mL, 4.29 mmol) was added dropwise and the solution stirred at room temperature under argon atmosphere overnight. The solvent was evaporated under reduced pressure, methyl 2-amino-4-(benzyloxy)benzo[*d*]thiazole-6-carboxylate (**9**, 270 mg, 0.859 mmol) and toluene (20 mL) were added and the suspension was stirred at 130 °C overnight. The precipitate in the reaction mixture was filtered off, suspended in 1 M HCl (100 mL), sonicated and filtered off. The crude product was dispersed in methanol (100 mL), heated, filtered off and dried. Yield: 311 mg (73.9%); grey solid. ^{1}H NMR (400 MHz, DMSO-d_6) δ 2.26 (s, 3H), 3.89 (s, 3H), 5.32 (s, 2H), 7.33-7.49 (m, 3H), 7.54 (m, 2H), 7.62 (d, *J* = 1.3 Hz, 1H), 8.29 (s, 1H), 12.19 (s, 1H), 12.22 (s, 1H).

4-(Benzyloxy)-2-(3,4-dichloro-5-methyl-1*H*-pyrrole-2-carboxamido)benzo[*d*]thiazole-6-carboxylic acid (11 (ULD2)). Methyl 4-(benzyloxy)-2-(3,4-dichloro-5-methyl-1*H*-pyrrole-2-carboxamido)benzo[*d*]thiazole-6-carboxylate (**10**, 100 mg, 0.204 mmol) was suspended in methanol (20 mL), 1 M NaOH (1.02 mL, 1.02 mmol) was added and the reaction mixture was stirred at 40 °C overnight. 500 µL of 1 M NaOH was added and the reaction mixture was stirred at 40 °C overnight. The

solvent was removed under reduced pressure, the residue was acidified with 1 M HCl to pH = 1, sonicated and the obtained precipitate was filtered off. The crude product was suspended in THF, sonicated, heated, filtered hot and the mother liquid evaporated under reduced pressure. To the dry residue methanol was added, sonicated, heated, and the precipitate was filtered out of a hot suspension. The precipitate was washed with THF and methanol and dried. Yield: 40 mg (41.2 %); off-white solid. ^{1}H NMR (400 MHz, DMSO-d_6) δ 2.26 (s, 3H), 5.32 (s, 2H), 7.57 – 7.35 (m, 5H), 7.61 (d, J = 1.3 Hz, 1H), 8.25 (d, J = 1.0 Hz, 1H), 12.16 (s, 1H), 12.23 (s, 1H), 13.00 (s, 1H) (S4 Fig). ^{13}C NMR (101 MHz, DMSO-d_6) δ 11.03, 70.02, 108.95, 109.91, 115.58, 116.26, 116.86, 126.72, 128.10, 128.30, 128.43, 129.95, 132.79, 136.59, 141.85, 150.11, 156.62, 159.58, 167.06 (S5 Fig). HRMS (ESI$^-$) *m/z* for $C_{21}H_{14}Cl_2N_3O_4S$ ([M-H]$^-$): calculated 474.0077, found 474.0095. HPLC: t_r 11.630 min (99.1 % at 254 nm) (S6 Fig).

^{1}H and ^{13}C NMR spectra and HPLC chromatograms for ULD1 and ULD2:

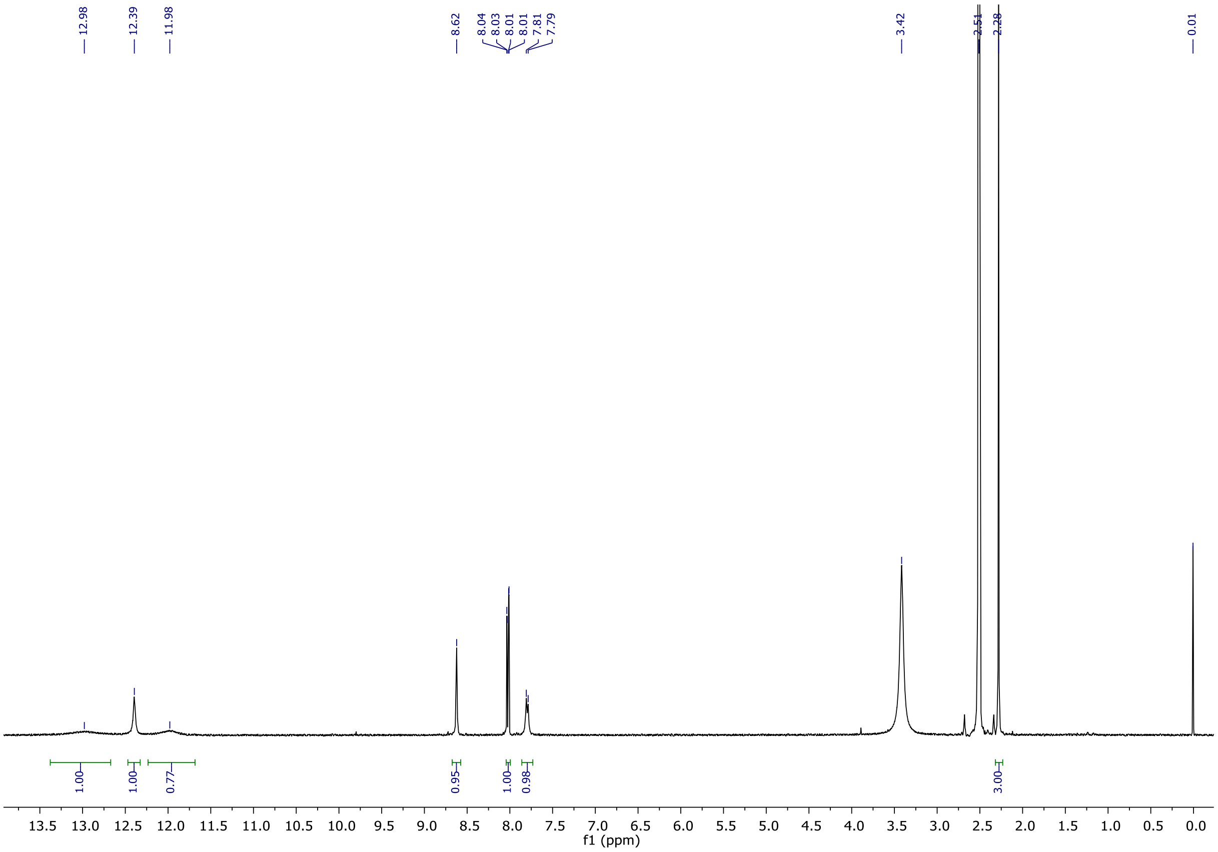

S1 Fig. ^{1}H NMR spectra of ULD1.

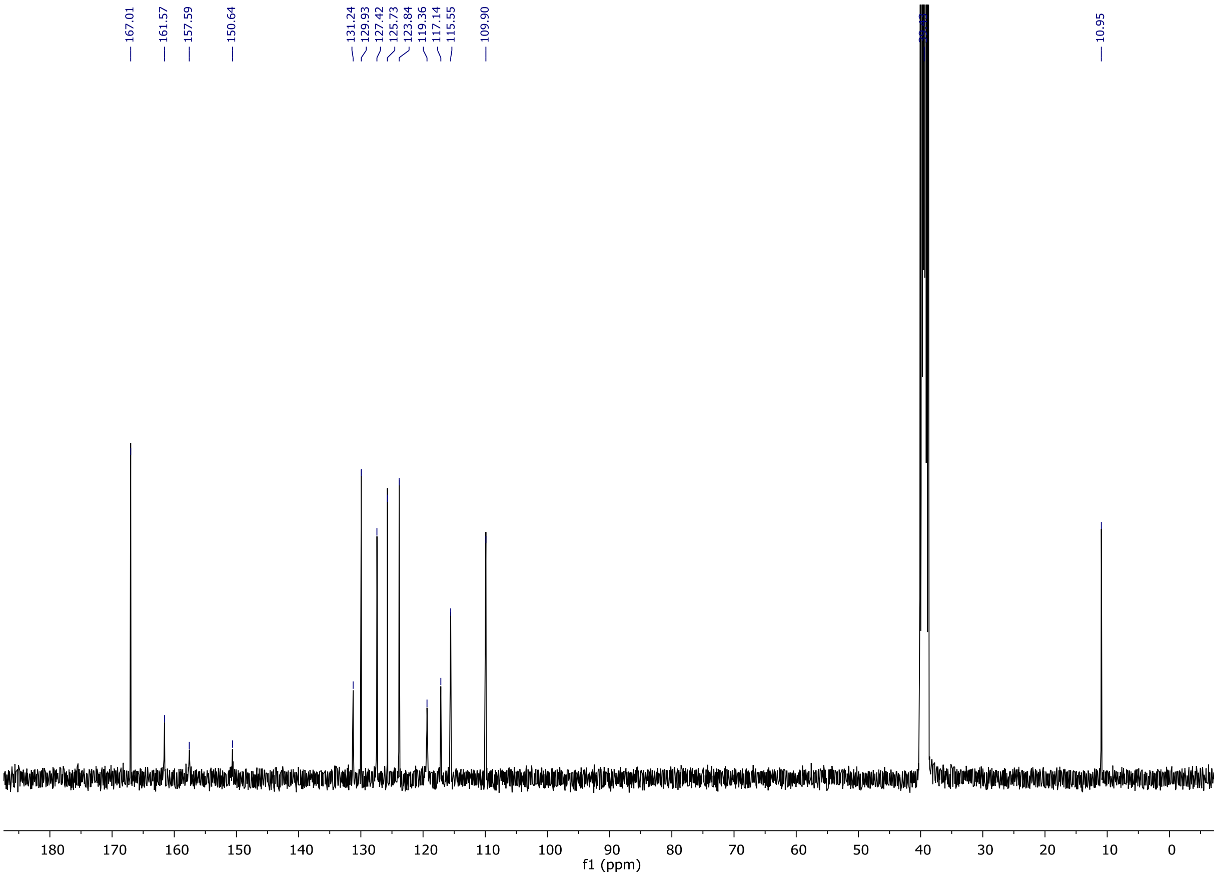

S2 Fig. ^{13}C NMR spectra of ULD1.

Chromatogram

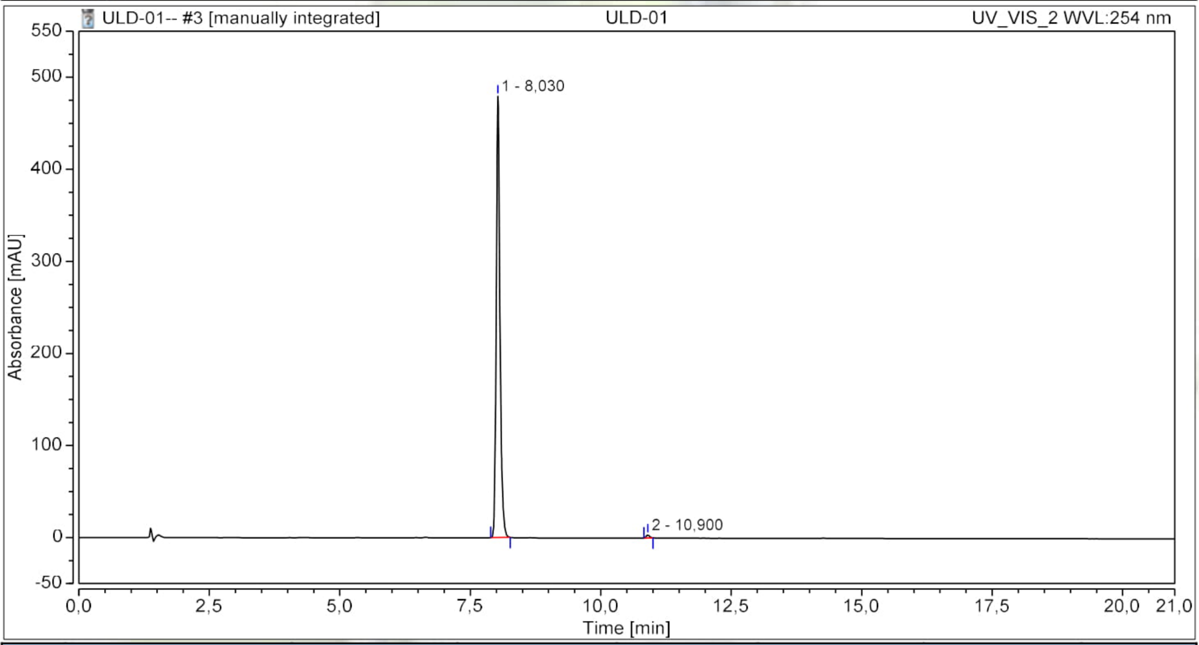

Integration Results

No.	Peak Name	Retention Time<br>min	Area<br>mAU*min	Height<br>mAU	Relative Area<br>%	Relative Height<br>%	Amount<br>n.a.
1		8,030	39,903	479,548	99,40	99,34	n.a.
2		10,900	0,243	3,180	0,60	0,66	n.a.
**Total:**			**40,145**	**482,727**	**100,00**	**100,00**	

S3 Fig. HPLC chromatogram of ULD1.

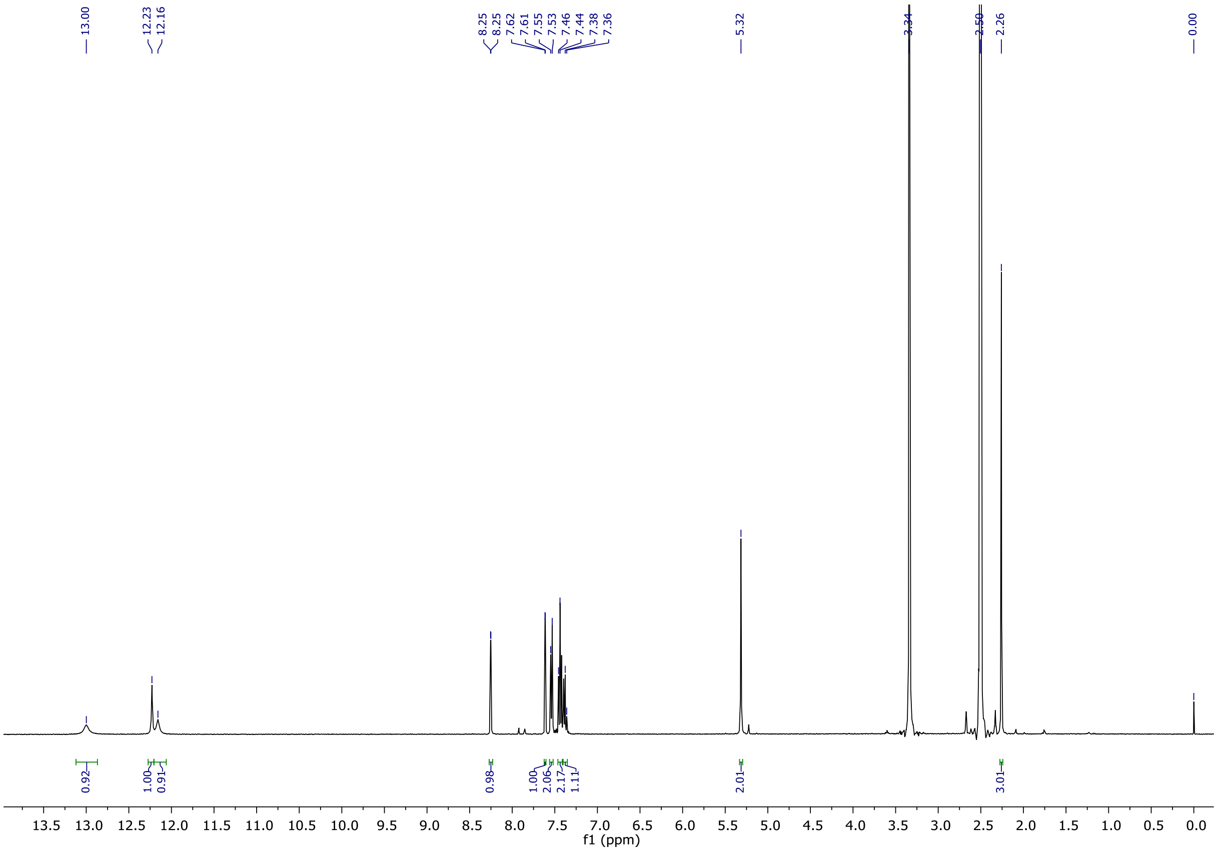

S4 Fig. ^{1}H NMR spectra of ULD2.

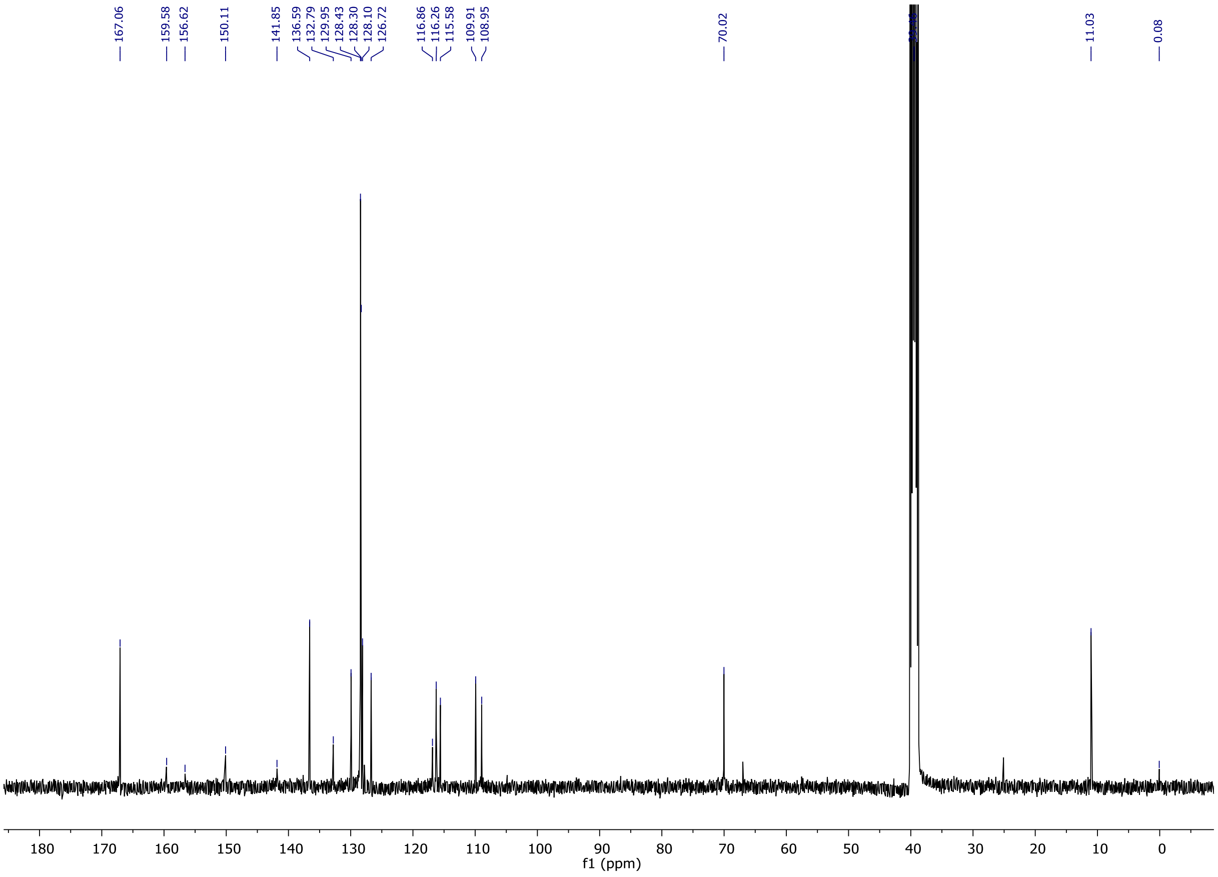

S5 Fig. ^{13}C NMR spectra of ULD2.

Chromatogram

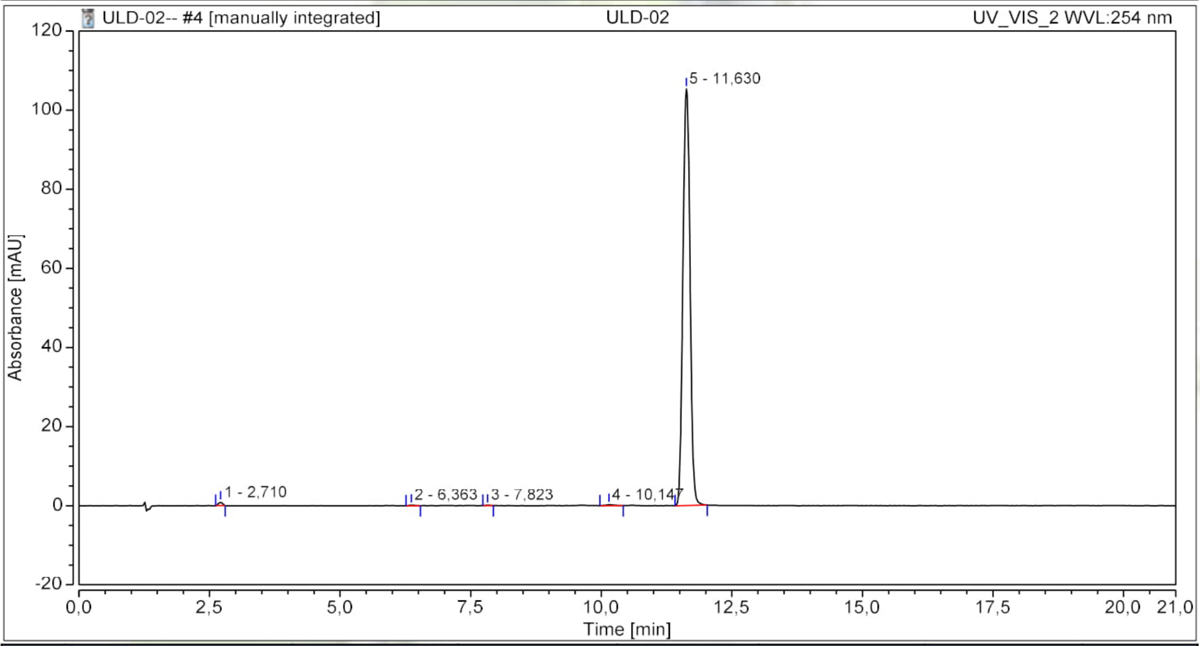

Integration Results

No.	Peak Name	Retention Time min	Area mAU*min	Height mAU	Relative Area %	Relative Height %	Amount n.a.
1		2,710	0,079	0,849	0,47	0,80	n.a.
2		6,363	0,018	0,127	0,11	0,12	n.a.
3		7,823	0,008	0,077	0,05	0,07	n.a.
4		10,147	0,050	0,253	0,30	0,24	n.a.
5		11,630	16,588	105,343	99,07	98,78	n.a.
**Total:**			**16,743**	**106,648**	**100,00**	**100,00**	

S6. Fig. HPLC chromatogram of ULD2.
